# Molecular characterization and expression of sensory neuron membrane proteins in the parasitoid *Microplitis mediator* (Hymenoptera: Braconidae)

**DOI:** 10.1111/1744-7917.12667

**Published:** 2019-03-14

**Authors:** Shuang Shan, Shan‐Ning Wang, Xuan Song, Adel Khashaveh, Zi‐Yun Lu, Khalid Hussain Dhiloo, Rui‐Jun Li, Xi‐Wu Gao, Yong‐Jun Zhang

**Affiliations:** ^1^ College of Plant Protection China Agricultural University Beijing China; ^2^ State Key Laboratory for Biology of Plant Diseases and Insect Pests, Institute of Plant Protection Chinese Academy of Agricultural Sciences Beijing China; ^3^ Institute of Plant and Environment Protection Beijing Academy of Agricultural and Forestry Sciences Beijing China; ^4^ College of Plant Protection Agricultural University of Hebei Baoding China; ^5^ IPM Center of Hebei Province, Key Laboratory of Integrated Pest Management on Crops in Northern Region of North China, Ministry of Agriculture, Plant Protection Institute Hebei Academy of Agricultural and Forestry Sciences Baoding China; ^6^ Department of Entomology, Faculty of Crop Protection Sindh Agriculture University Tandojam Pakistan

**Keywords:** antennae, chemosensation, expression profile, *in situ* hybridization, *Microplitis mediator*, sensory neuron membrane proteins

## Abstract

Sensory neuron membrane proteins (SNMPs), homologs of the human fatty acid transport protein CD36 family, are observed to play a significant role in chemoreception, especially in detecting sex pheromone in *Drosophila* and some lepidopteran species. In the current study, two full‐length SNMP transcripts, *MmedSNMP1* and *MmedSNMP2*, were identified in the parasitoid *Microplitis mediator* (Hymenoptera: Braconidae). Quantitative real‐time polymerase chain reaction analysis showed that the expression of *MmedSNMP1* was significantly higher in antennae than in other tissues of both sexes. In addition, the *MmedSNMP1* transcript was increased dramatically in newly emerged adults and there were no significant differences between adults with or without mating and parasitic experiences. However, compared with *MmedSNMP1*, the expression of *MmedSNMP2* was widely found in various tissues, significantly increased at half‐pigmented pupae stage and remained at a relatively constant level during the following developmental stages. It was found that *MmedSNMP1* contained eight exons and seven introns, which was highly conserved compared with other insect species. *In situ* hybridization assay demonstrated that *MmedSNMP1* transcript was distributed widely in antennal flagella. Among selected chemosensory genes (odorant binding protein, odorant receptor, and ionotropic receptor genes), *MmedSNMP1* only partially overlapped with *MmedORco* in olfactory sensory neurons of antennae. Subsequent immunolocalization results further indicated that MmedSNMP1 was mainly expressed in sensilla placodea of antennae and possibly involved in perceiving plant volatiles and sex pheromones. These findings lay a foundation for further investigating the roles of SNMPs in the chemosensation of parasitoids.

## Introduction

Chemoreception contributes enormously to the survival and reproduction of insects. As for insects, they rely basically on their sensitive antennae to perceive semiochemicals in the complex environment (Ache & Young, 2005; Su *et al*., 2009; Sachse & Krieger, 2011; Leal, 2013). Over the last decade, significant progress has been made to understand the peripheral molecular mechanism responsible for the recognition of olfactory signals in the insect antennae (Sachse & Krieger, 2011). In insect olfactory perception, odorant molecules penetrate the cuticular pores in sensilla of antennae, and then odorants are detected and transmitted by odorant binding proteins (OBPs) or chemosensory proteins (CSPs) in the sensilla lymph. Subsequently, receptor proteins such as odorant receptors (ORs) and ionotropic receptors (IRs) situated in the dendritic membrane of olfactory sensory neurons (OSNs) are activated by the ligand‐binding protein complexes or odorant molecules alone (Vogt & Riddiford, 1981; Steinbrecht, 1997; Wanner *et al*., 2004; Benton *et al*., 2009; Kaissling, 2009; Sánchez‐Gracia *et al*., 2009; Zhou, 2010; Leal, 2013). In addition, the sensory neuron membrane proteins (SNMPs), another olfactory protein family in the peripheral olfactory system of insects, are suggested to play an important role in odorant perception (Rogers *et al*., 2001a,b; Vogt, 2003; Benton *et al*., 2007).

Insect SNMPs, transmembrane proteins, are homologs of the vertebrate CD36 family, which are involved in recognizing and transporting hydrophobic molecules such as fatty acids and lipid‐protein complexes (Rogers *et al*., 1997; Ge & Elghetany, 2005; Benton *et al*., 2007; Jin *et al*., 2008; Silverstein & Febbraio, 2009). In insects, two SNMP subfamilies, SNMP1 and SNMP2, have been identified. As we known, SNMP1s are mainly expressed in adult antennae of some lepidopteran insects (Rogers *et al*., 1997, 2001a; Gu *et al*., 2013). SNMP1s are associated with pheromone‐specific neurons in some dipteran and lepidopteran insects, suggesting their roles in pheromone detection (Rogers *et al*., 1997, 2001a,b; Benton *et al*., 2007; Jin *et al*., 2008; Pregitzer *et al*., 2014; Zielonka *et al*., 2018). For instance, it was suggested that DmelSNMP1 of *Drosophila* is required in detecting 11‐*cis*‐vaccenyl acetate (cVA), a male‐specific pheromone. Some scholars proposed that DmelSNMP1 could interact directly with the cVA receptor protein OR67d (Benton *et al*., 2007), or act as an inhibitory subunit in a complex with OR67d (Jin *et al*., 2008). Intriguingly, not being limited to expression in OSNs, SNMP1 was also found in the support cells throughout the antennae of *D. melanogaster* (Benton *et al*., 2007). Additionally, an interaction model was proposed that BmorORco, BmorSNMP1 and BmorOR1 formed a heteromer in detection of silkworm sex pheromone bombykol in *Bombyx mori* (Zhang *et al*., 2018). Compared with SNMP1s, SNMP2s are mostly expressed in support cells or sensillum lymph surrounding the pheromone‐sensitive sensilla neurons in some lepidopteran species such as *Heliothis virescens*, *Antheraea polyphemus*, *Agrotis ipsilon* and *Spodoptera litura* as well as orthopteran *Schistocerca gregaria* (Rogers *et al*., 2001b; Forstner *et al*., 2008; Gu *et al*., 2013; Zhang *et al*., 2015; Jiang *et al*., 2016). The distinct expression profiles imply that SNMP1 and SNMP2 are likely to perform diverse functions in chemoreception. Recently, a novel subfamily, SNMP3, was identified in Lepidoptera, which may play roles in immunity response of insects (Zhang *et al*., 2018).

*Microplitis mediator* (Haliday) (Hymenoptera: Braconidae), a widely distributed generalist endoparasitoid wasp in Asia and Europe, attacks approximately 40 different lepidopteran larvae (Arthur & Mason, 1986; Khan, 1999; Mason *et al*., 2001). In North China, *M. mediator* is an important natural enemy of target insects and has been employed as a biocontrol agent to prevent the infestation of *H. armigera* (Li *et al*., 2006). Like most other parasitoid wasps, *M. mediator* utilizes olfaction to detect host‐related chemical cues for habitat searching, host location and assessment. The morphological characteristics of antennal sensilla, including three types of s. basiconica, s. trichodea, s. placodea, s. chaetica, s. coeloconica and s. campaniform in *M. mediator* were fully described; all of these sensilla were also identified in other hymenopteran insects (Ochieng *et al*., 2000; Gao *et al*., 2007; Meng *et al*., 2012; Ahmed *et al*., 2013; Huang *et al*., 2018; Wang *et al*., 2018). In our previous works, large sets of chemoreceptors (169 ORs, 17 IRs and two gustatory receptors) and small soluble proteins (18 OBPs, three CSPs and two Niemann‐Pick type C2 proteins) in antennae of *M. mediator* were characterized (Zhang *et al*., 2009, 2011; Li *et al*., 2014; Ma *et al*., 2014; Wang *et al*., 2015, 2016, 2017; Peng *et al*., 2017; Zheng *et al*., 2018). However, little is known about the different roles of SNMPs in chemoreception of *M. mediator* and other wasps.

In the present study, two SNMP genes, *MmedSNMP1* and *MmedSNMP2*, were identified from antennae of *M. mediator*. The temporal and spatial expression patterns of *MmedSNMP1* and *MmedSNMP2* among different tissues along development stages of both sexes were evaluated by quantitative real‐time polymerase chain reaction (qPCR) analysis. Subsequent studies were focused on MmedSNMP1. The distribution of *MmedSNMP1* was investigated extensively by *in situ* hybridization assays. Moreover, the fluorescence immunocytochemistry experiment was performed to locate the MmedSNMP1 in antennae. Our data will provide new insights to explore the role of SNMPs in chemical communication of *M. mediator*.

## Materials and methods

### Insect rearing and tissues collection

The cocoons of *M. mediator* were reared in an artificial climate incubator with a condition of 28 ± 1°C, 60% ± 10% RH (relative humidity) and 16 : 8 L : D photoperiod. The newly emerged adults were fed on 10% sucrose solution. In the SNMP genes cloning and expression analysis, different tissues (male antennae, female antennae, heads without antennae, thoraxes, abdomens, legs, wings) from 2‐ or 3‐day‐old adult wasps were collected. The antennae were dissected from the female and male wasps at different developmental stages (red‐eyed stage, half‐pigmented stage, fully pigmented stage, 1 day after emergence) and distinct physiological states (3‐day‐old virgins, 3‐day‐old mated wasps with or without parasitic experience). For males, the parasitic experience means that mated males were reared in a small space together with their hosts. All tissues were immediately frozen in liquid nitrogen and stored at –80°C until use.

### Total RNA isolation and cDNA synthesis

Total RNA was isolated by using Trizol reagent (Invitrogen, Carlsbad, CA, USA), after which the integrity was checked by using 1.1% agarose gel electrophoresis. The extracted RNA was quantified on a ND‐2000 spectrophotometer (NanoDrop, Wilmington, DE, USA). Then the total RNA was treated with RQ1 RNase‐Free DNase (Promega, Madison, WI, USA) to remove residual genomic DNA. In SNMP genes cloning and qPCR analysis, 1 *μ*g of the total RNA was employed to synthesize the first‐stranded complementary DNA (cDNA) using the SuperScript^TM^ III Reverse Transcriptase kit (Invitrogen, Carlsbad, CA, USA).

### Identification of SNMP genes and sequence analysis

Full‐length sequences of putative *MmedSNMP1* and *MmedSNMP2* were obtained from previous antennal transcriptome data of *M. mediator* (Wang *et al*., 2015, 2016). The gene‐specific primers (Table S1) were designed by Primer Express 3.0 (Applied Biosystems, Carlsbad, CA, USA) to amplify the open reading frame (ORF) of *MmedSNMP1* and *MmedSNMP2*. PCR conditions were as follows: an initial denaturation at 95°C for 5 min; followed by 38 cycles of 95°C for 30 s, 55°C for 30 s and 72°C for 2 min; and then a final extension at 72°C for 10 min. The PCR products were cloned into the pEasy‐T3 vector (TransGen, Beijing, China) and then sequenced.

In phylogenetic analysis, SNMP1‐2 sequences from 28 insect species (Supplemental material 1) were compared using BLASTX (http://www.ncbi.nlm.nih.gov/). Amino acid sequences were aligned using ClustalX 2.1 (Larkin *et al*., 2007) and edited by ESPript 3.0 (http://espript.ibcp.fr) (Robert & Gouet, 2014). A neighbor‐joining tree of SNMP orthologs from various insect species was constructed using MEGA 7.0 software (Kumar *et al*., 2016) with a *p*‐distance model and pairwise deletion of gaps. Bootstrap support of tree branches was assessed by re‐sampling amino acid positions 1000 times. Transmembrane domain predictions were performed using TMHMM Server v. 2.0 (http://www.cbs.dtu.dk/services/TMHMM-2.0/), and topology structures were constructed using TOPO2 Transmembrane Protein Display (http://www.sacs.ucsf.edu/cgi-bin/open-topo2.py/).

### qPCR measurement

The expression profiles of *MmedSNMP1‐2* were evaluated by qPCR analysis on an ABI Prism 7500 Fast Detection System (Applied Biosystems, Carlsbad, CA, USA). The reference gene *β‐actin* (GenBank accession number: KC193266.1) was used as the endogenous control to normalize the target gene expression and correct for any sample‐to‐sample variation. The primers (Table S1) of target and reference genes were designed by Beacon Designer 7.0 (PREMIER Biosoft International, Palo Alto, CA, USA). The specificity of each primer set was validated by melt‐curve analysis, and the efficiency was calculated by analyzing standard curve with a five‐fold cDNA dilution series. Each qPCR reaction was conducted in a 20 *μ*L reaction mixture containing 10 *μ*L of SuperReal PreMix Plus (TIANGEN, Beijing, China), 1 *μ*L of sample cDNA (200 ng), 0.6 *μ*L of sense and antisense primer (10 *μ*mol/L), 0.4 *μ*L of Rox Reference Dye and 7.4 *μ*L of sterilized H_2_O. The qPCR cycling parameters consisted of 95°C for 15 min, followed by 40 cycles of 95°C for 10 s, 55°C for 30 s, 72°C for 32 s, and melt curve stage at 95°C for 15 s, 60°C for 1 min, and 95°C for 15 s. The experiments for the test samples, endogenous control and negative control were performed in triplicate to ensure reproducibility. The comparative $2^{-\Delta \Delta C_{T}}$ method was used to calculate the relative transcript level of the target gene in each sample (Livak & Schmittgen, 2001). Datas from qPCR tests were analyzed using SPSS Statistics 17.0 (SPSS Inc., Chicago, IL, USA). Analysis of variance and Duncan's multiple range test (*P* < 0.05) were used to determine whether differences in *MmedSNMP* messenger RNA levels were significant among different tissue samples. The *t*‐test was employed to evaluate differences in SNMPs of the same tissue samples between males and females.

### MmedSNMP1 gene structure analysis

Genomic DNA of *M. mediator* was extracted using TIANamp genomic DNA kit (TIANGEN, Beijing, China) following the manufacturer's instruction. Introns of *MmedSNMP1* were amplified using specific primers (Table S1). The gene structure was analyzed using GSDS 2.0 (Hu *et al*., 2015) and Splign (https://www.ncbi.nlm.nih.gov/sutils/splign/). The ClustalX 2.1 alignments of *SNMP1* sequences from hymenopteran, dipteran, lepidopteran and coleopteran species (Supplemental material 2) were used to character intron insertion sites.

### In situ hybridization

Digoxigenin (DIG)‐labeled and biotin‐labeled antisense or sense RNA probes were generated from linearized recombinant plasmids containing the coding region of target genes using the DIG RNA Labeling Kit (SP6/T7) and Biotin RNA Labeling Mix (Roche, Mannheim, Germany). Specific primers were designed to amplify the target gene sequences (Table S1). The labeled probes were fragmented to an average length of about 400 bp by incubation in carbonate buffer (80 mmol/L NaHCO_3_, 120 mmol/L Na_2_CO_3_, pH 10.2) following the protocol of Cox *et al*. (1984).

Antennae were embedded in Tissue‐Tek optimal cutting temperature (OCT) compound (Sakura Finetek, Torrance, CA, USA) and cut into 12 *μ*m slices at −26°C by Cryostar NX50 freezing microtome (Thermo Scientific, San Jose, CA, USA). Sections were pasted on Superfrost plus microscope slides (Fisher Scientific, Pittsburgh, PA, USA) and stored at −80°C until use. Hybridization was performed based on previous reports (Yang *et al*., 2012; Xu *et al*., 2013; Guo *et al*., 2014; Xu *et al*., 2017). Briefly, slides of antennae were dried at room temperature for 30 min and fixed in 4% paraformaldehyde solution at 4°C for 30 min, then were incubated in 0.2 mol/L HCl for 10 min and washed in phosphate‐buffered saline (PBS). Slides were pre‐hybridized for 1 h in 50% formamide with 2 × saline‐sodium citrate (SSC) solution. One hundred microliters of hybridization solution containing probe of target gene was added to the corresponding slides and then slides were incubated at 60°C for at least 16 h. After hybridization, slides were washed three times in 0.1 × SSC at 60°C for 20 min, and then incubated in 1% blocking reagent (Roche, Mannheim, Germany) diluted in Tris‐buffered saline (100 mmol/L Tris, 150 mmol/L NaCl, pH 7.5) with 0.03% Triton X‐100 at room temperature for 30 min. DIG‐labeled probe was detected by anti‐DIG alkaline phosphatase conjugated antibody (Roche, Mannheim, Germany) combined with 2‐hydroxy‐3‐naphtoic acid‐2'‐phenylanilide phosphate (HNPP) substrate (Roche, Mannheim, Germany). For the biotin‐labeled probe, Strepavidin‐HRP (horseradish peroxidase) and TSA (tyramide signal amplification) Kit (Perkin Elmer, Boston, MA, USA) were employed to detect signals. Tissue sections were observed using a Zeiss LSM 880 confocal microscope (Carl Zeiss Microscopy GmbH, Jena, Germany) and images were processed with ZEN 2 (Carl Zeiss Microscopy GmbH, Jena, Germany).

### Western blot assay

The rabbit antiserum against a synthetic peptide of MmedSNMP1 (amino acid sequence: GILREDDSGFLKDG) was produced by GenScript Biotech (Nanjing, China). Crude antennal proteins were extracted using Trizol (Invitrogen, Carlsbad, CA, USA). Protein samples were separated by 15% SDS‐PAGE and then transferred to a polyvinylidene fluoride membrane (PVDF) (Millipore, Carrigtwohill, Ireland). The membrane was blocked using 5% fat‐free milk (BD Biosciences, San Jose, CA, USA) in PBS containing 0.05% Tween‐20 (PBST) at 4°C overnight. After washing three times with PBST (10 min each), the blocked membrane was incubated with rabbit anti‐MmedSNMP1 antiserum (1 : 4000) at room temperature for 1 h. After three additional washes with PBST, the membrane was incubated with goat anti‐rabbit immunoglobulin G (IgG) HRP‐conjugated antibody (GenScript, Nanjing, China) (1 : 5000) at room temperature for 2 h. Finally, the membrane was developed using Easy‐See Western Blot kit (TransGen, Beijing, China), then exposed and imaged on an Image‐Quant LAS 4000 mini (GE Healthcare Bio‐Sciences AB, Uppsala, Sweden).

### Immunocytochemical localization

Male antennae were dissected and pre‐fixed in 4% paraformaldehyde at 4°C for 30 min, then transferred to 25% sucrose solution at 4°C overnight. After pretreatment, antennae were cut into 12 *μ*m slices. Tissue sections were allowed to dry at room temperature for 1 h and fixed in 4% paraformaldehyde at 4°C for 30 min. Subsequently, samples were washed three times with PBST and blocked using 5% normal goat serum (Jackson ImmunoResearch, West Grove, PA, USA) in PBST for 1 h at room temperature. After rinsing, sections were incubated with anti‐MmedSNMP1 antiserum (1 : 4000) at 4°C overnight. Next day, samples were treated with goat anti‐rabbit IgG‐conjugated Alexa Fluor 488 (Invitrogen, Carlsbad, CA, USA) (1 : 500) and mounted in mowiol solution (10% mowiol 4‐88, 20% glycerol in PBS). Finally, a Zeiss LSM 880 confocal microscope (Carl Zeiss Microscopy GmbH, Jena, Germany) was used to observe tissue sections and capture images.

## Results

### Identification of SNMPs in M. mediator

Two full‐length SNMP transcripts were successfully obtained from an antennae transcriptome database and named as *MmedSNMP1* (GenBank accession number: KM245938.1) and *MmedSNMP2* (GenBank accession number: MH229861), respectively. The ORFs of *MmedSNMP1* and *MmedSNMP2* are 1578 bp and 1377 bp in length encoding 525 and 459 amino acids, separately. The calculated molecular weights of MmedSNMP1‐2 proteins are 58.48 kDa and 52.57 kDa, and the isoelectric points are 5.98 and 7.04, respectively. Both MmedSNMP1 and MmedSNMP2 are characterized by a general structure shared with members of the CD36 receptor family, which appear as two transmembrane domains with the N‐terminus being located inside the cell and a single large extracellular loop (Fig. S1).

### MmedSNMPs sequence analysis

**Figure 1:**
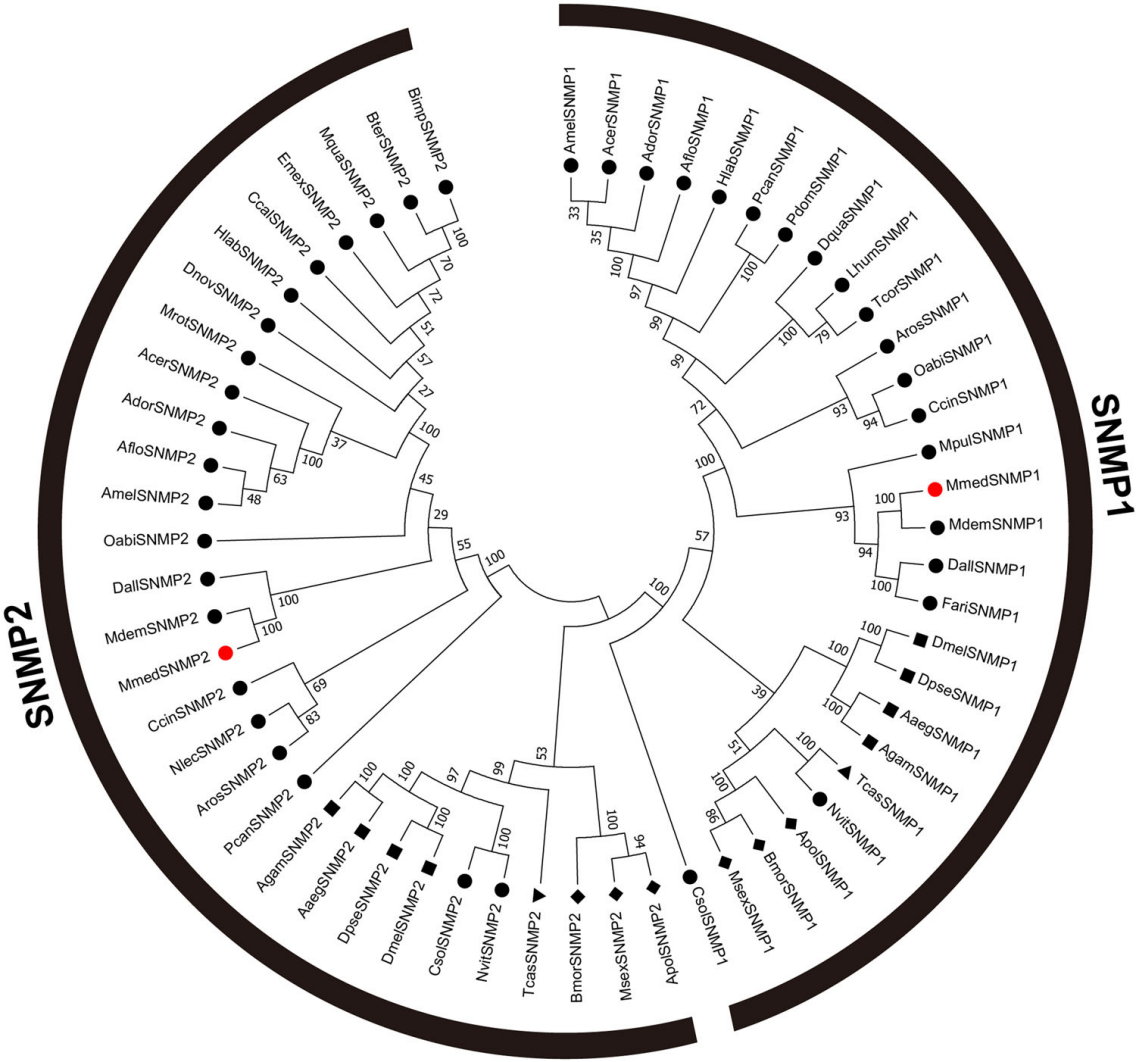
Phylogenetic analysis of sensory neuron membrane proteins (SNMPs) from different insect orders. This neighbor‐joining tree is constructed using MEGA 5 with a *p*‐distance model and pairwise by resampling amino acid position 1000 times. The SNMP sequences used in this analysis are listed in Supplemental material 1.

The phylogenetic analysis indicated that all selected proteins could be assigned to two distinct subfamilies, SNMP1 and SNMP2 (Fig. 1), with an order‐specific sequence cluster manner. MmedSNMP1‐2 located to the same branch, which consisted of SNMP1‐2 from hymenopteran species. MmedSNMP1‐2 were clustered closely with SNMP1‐2 of *M. demolitor*, which showed 100% homology in each branch. In addition, relatively high amino acid identities were found within the SNMP1 and SNMP2 subfamilies from hymenopteran species (59.12% identity in SNMP1 subfamily and 41.68% identity in SNMP2 subfamily) (Fig. S2).

### Expression pattern of MmedSNMPs

**Figure 2:**
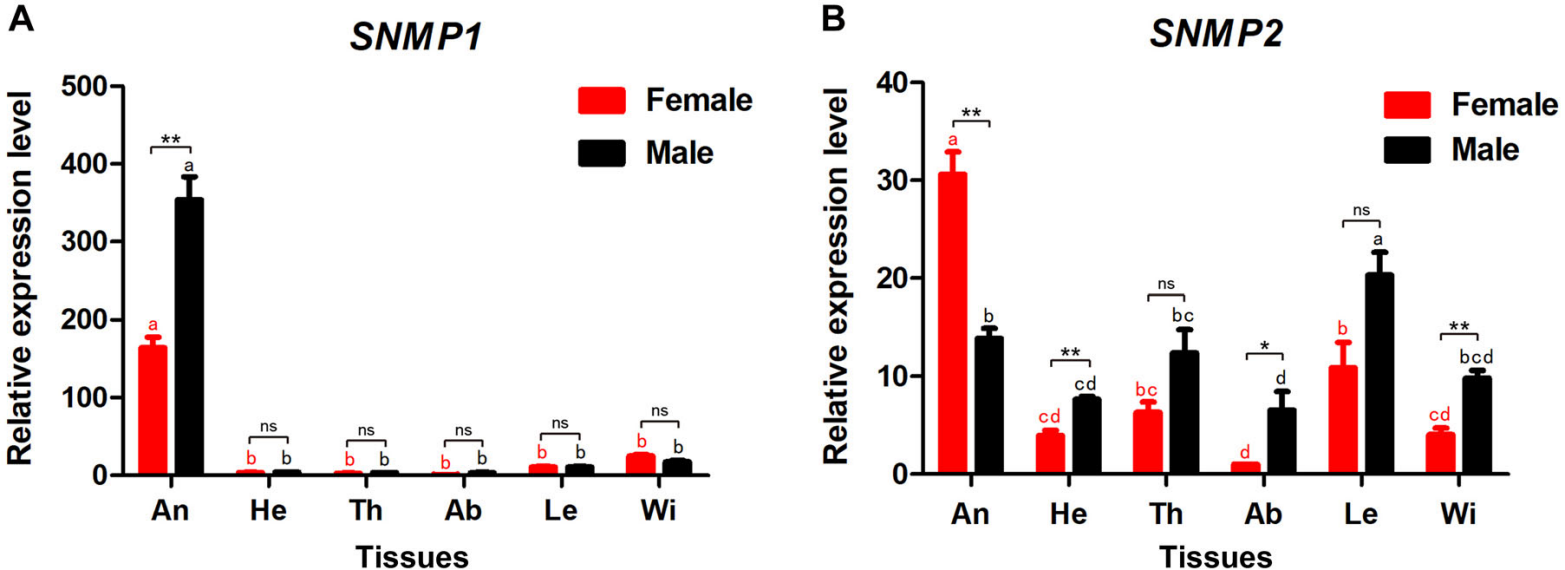
Expression profiles of *MmedSNMP1* (A) and *MmedSNMP2* (B) in different tissues. An: antennae, He: heads, Th: thoraxes, Ab: abdomens, Le: legs, Wi: wings. The fold changes are relative to the transcript levels in the abdomens of both sexes. The different small letters above each bar indicate significant differences in *SNMP* transcript levels of different tissues (*P* < 0.05). The asterisks represent significant differences in expression levels of *SNMP*s between males and females. (*, 0.01 < *P* < 0.05; **, 0.001 < *P* < 0.01; ***, 0.0001 < *P* < 0.001, ns, no significant difference).

The qPCR results showed that *MmedSNMP1* was expressed significantly higher in antennae of both sexes than in other tissues (heads, thoraxes, abdomens, legs, wings; *P* < 0.05). Specifically, the transcription level of *MmedSNMP1* in antennae was about 98‐fold that in thoraxes of males and about 164‐fold that in abdomens of females (Fig. 2A). *MmedSNMP2* was widely expressed in each tested tissue, while being expressed relatively higher in antennae than in other tissues of females (Fig. 2B). Furthermore, the expression of *MmedSNMP1* in male antennae was about 2.1 times higher than in females, whereas the transcription of *MmedSNMP2* in female antennae was 2.2 times higher than in males (Fig. 2).

**Figure 3:**
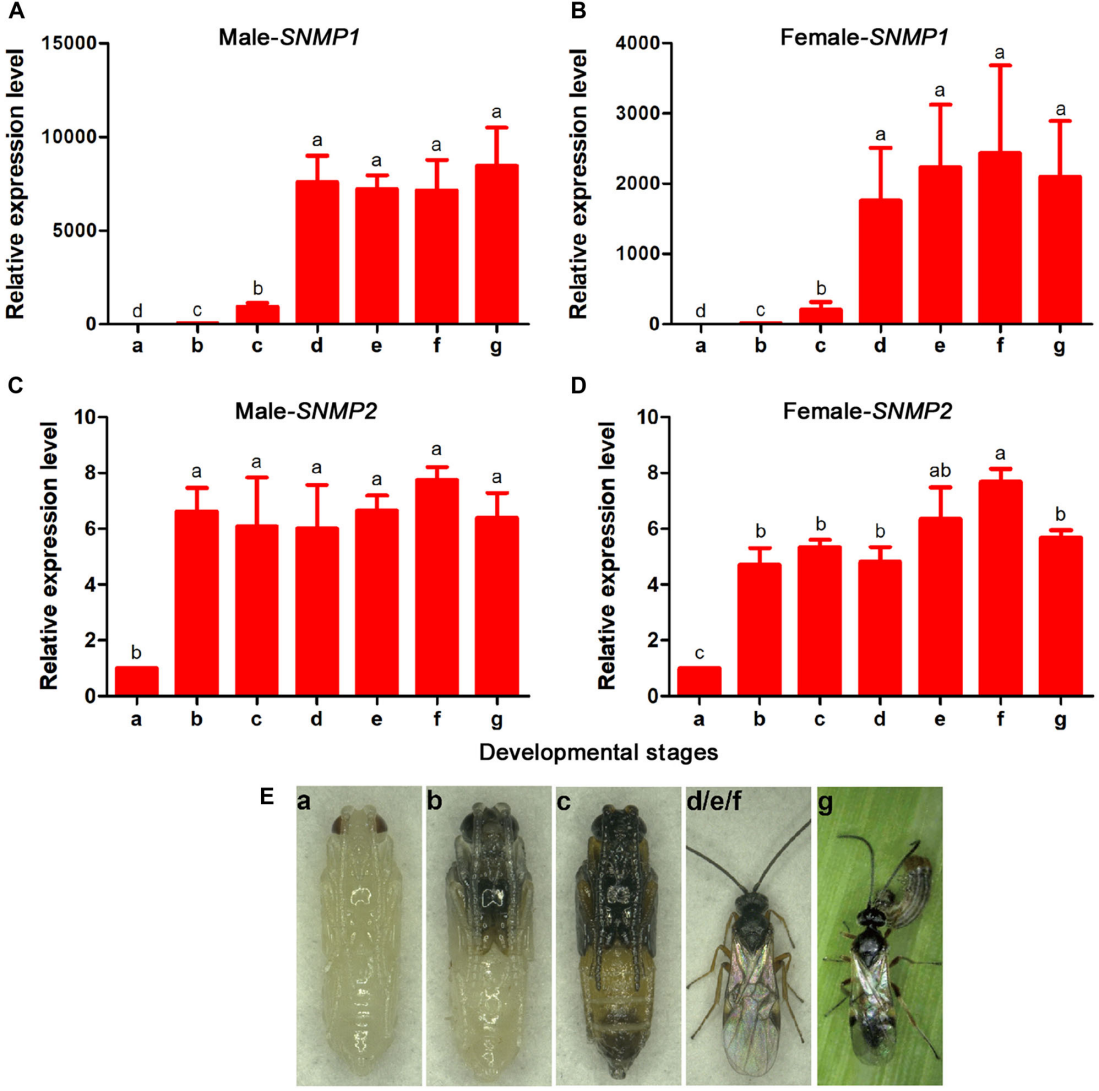
Expressions of *MmedSNMP1* and *MmedSNMP2* at different developmental stages and physiological states. (A–D) The fold changes are relative to the sensory neuron membrane protein (SNMP) transcript levels in antennae of *Microplitis mediator* at red‐eyed stage. The different small letters above each bar indicate significant differences in transcript abundances using Duncan's multiple range test (*P* < 0.05). (E) Schematic photos of *M. mediator* at different developmental stages and experience states. (a) Red‐eyed stage, (b) half‐pigmented stage, (c) fully pigmented stage, (d) newly emerged adult, (e) unmated adult, (f) mated adult without parasitic experience, (g) mated adult with parasitic experience.

The expressions of *MmedSNMP1* and *MmedSNMP2* in both sexes were developmentally regulated. At pupal stage of both sexes, a very low *MmedSNMP1* expression was detected within 3 days prior to the adult eclosion, while there was a strikingly increased expression at the emergence day (Fig. 3A, B). Compared with *MmedSNMP1*, the *MmedSNMP2* expression increased significantly at half‐pigmented stage and maintained a constant level during the subsequent developmental stages. Moreover, mating or parasitic experiences had no obvious effects on expression levels of *MmedSNMP1* and *MmedSNMP2* beside a slight increase of *MmedSNMP2* transcript in females with mating experience (Fig. 3C, D).

### Gene structure of MmedSNMP1

**Figure 4:**
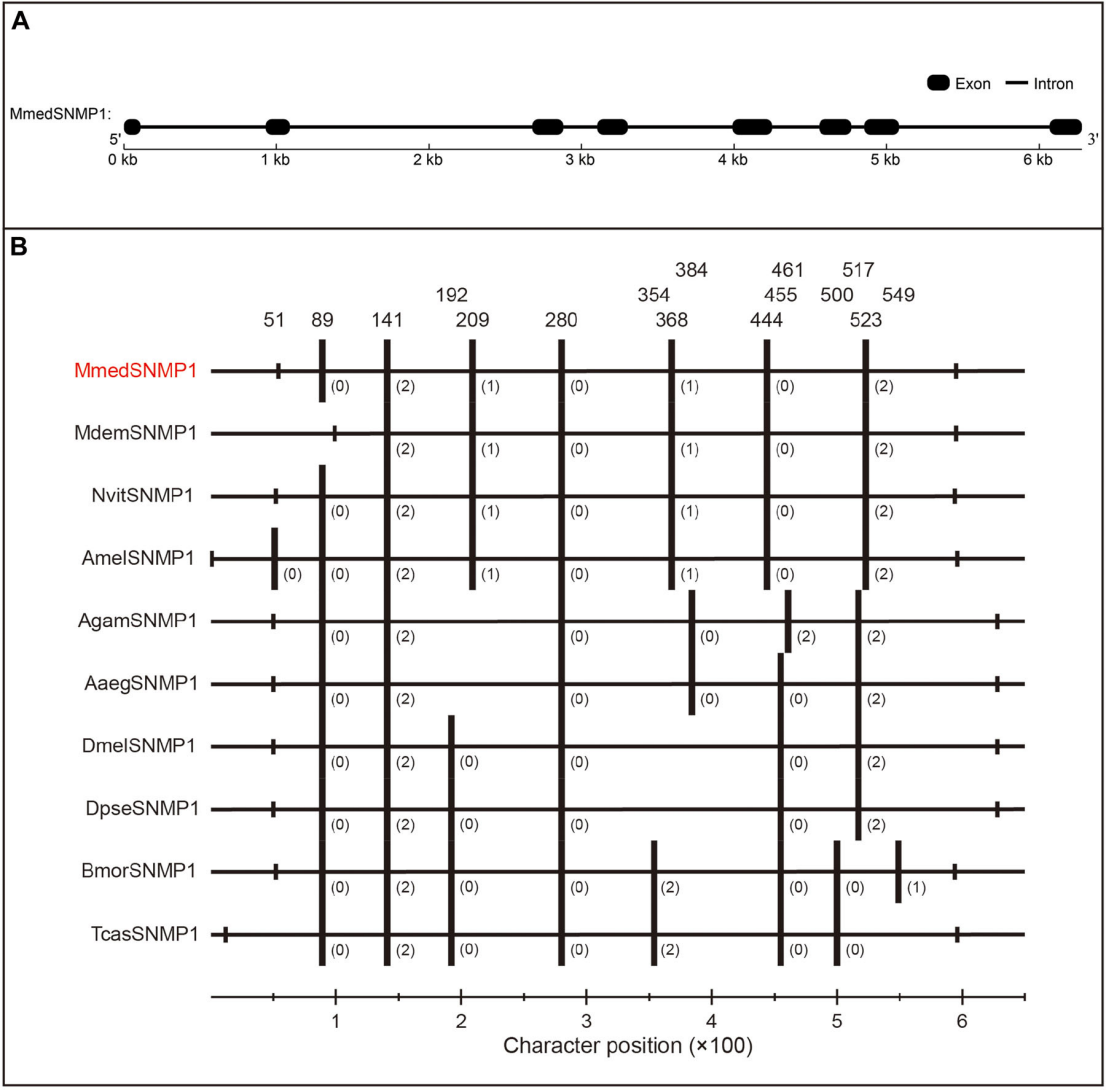
Gene structure and intron insertion sites analysis of sensory neuron membrane protein 1 (SNMP1). (A) Gene structure of *MmedSNMP1*. Exons are showed by black round‐corner rectangle and introns are showed by black line. (B) Comparison of intron insertion sites of SNMP1s among selected insect species. Long vertical bars show the introns insert position, short vertical bars show the initial and final amino acid positions. Numbers above indicate the insertion site character positions, while numbers in parentheses indicate the phase of each intron insertion site. Mmed: *Microplitis mediator*; Mdem: *M. demolitor*; Nvit: *Nasonia vitripennis*; Amel: *Apis mellifera*; Dmel: *Drosophila melanogaster*; Dpse: *D. pseudoobscura*; Aaeg: *Aedes aegypti*; Agam: *Anopheles gambiae*; Bmor: *Bombyx mori*; Tcas: *Tribolium castaneum*.

It was found that the genomic sequence size of MmedSNMP1 is 6279 bp containing eight exons and seven introns (Fig. 4A, Supplemental material 3). An amino acid alignment of SNMP1 from the Hymenoptera, Diptera, Lepidoptera and Coleoptera species is represented in Fig. 4B, showing only the positions of intron insertion sites and their phases (a codon not split by the intron has phase 0, a split codon has phase 1 or 2 depending on whether the split is between nucleotides 1–2 or nucleotides 2–3). Many intron insertion sites were clearly conserved across the family in all 10 species and all homologous intron insertion sites presented the same phases, suggesting the evolutionary relatedness between these genes.

### Localization of MmedSNMP1 in antennae of M. mediator

**Figure 5:**
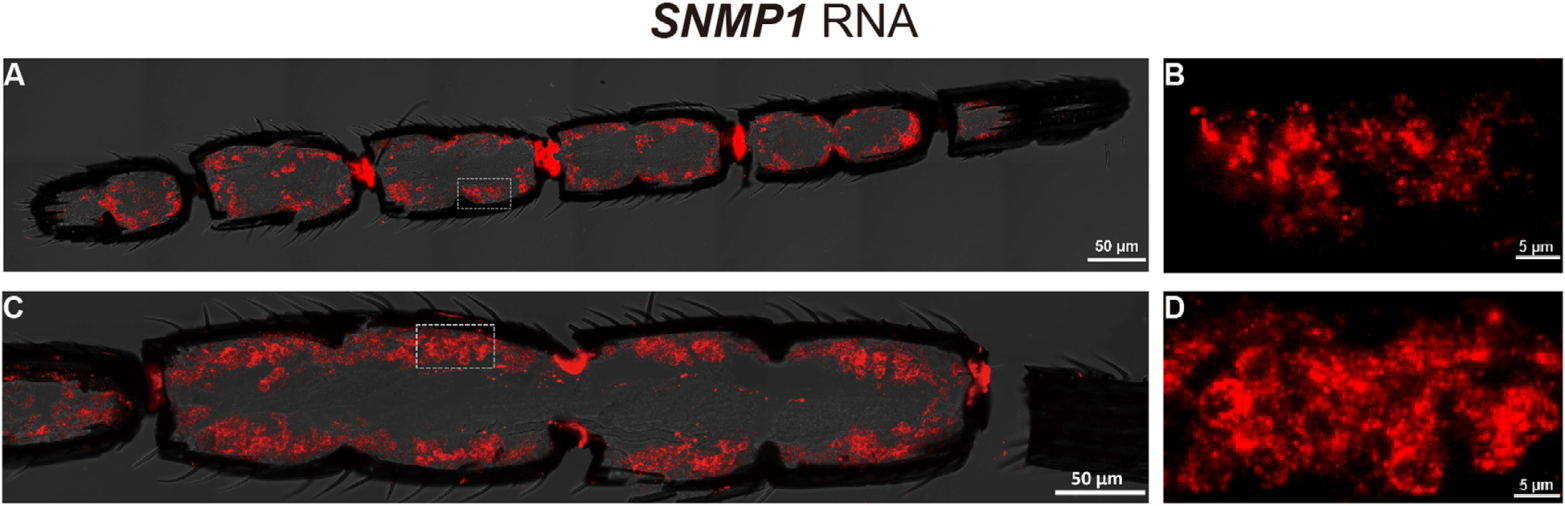
*In situ* hybridization assay of *SNMP1* in antennae of *Microplitis mediator*. Digoxigenin‐labeled antisense RNA probes for *SNMP1* are hybridized to cell clusters in longitudinal sections of antennal flagella segments of female (A, B) and male (C, D). Signals are visualized by red fluorescence.

**Figure 6:**
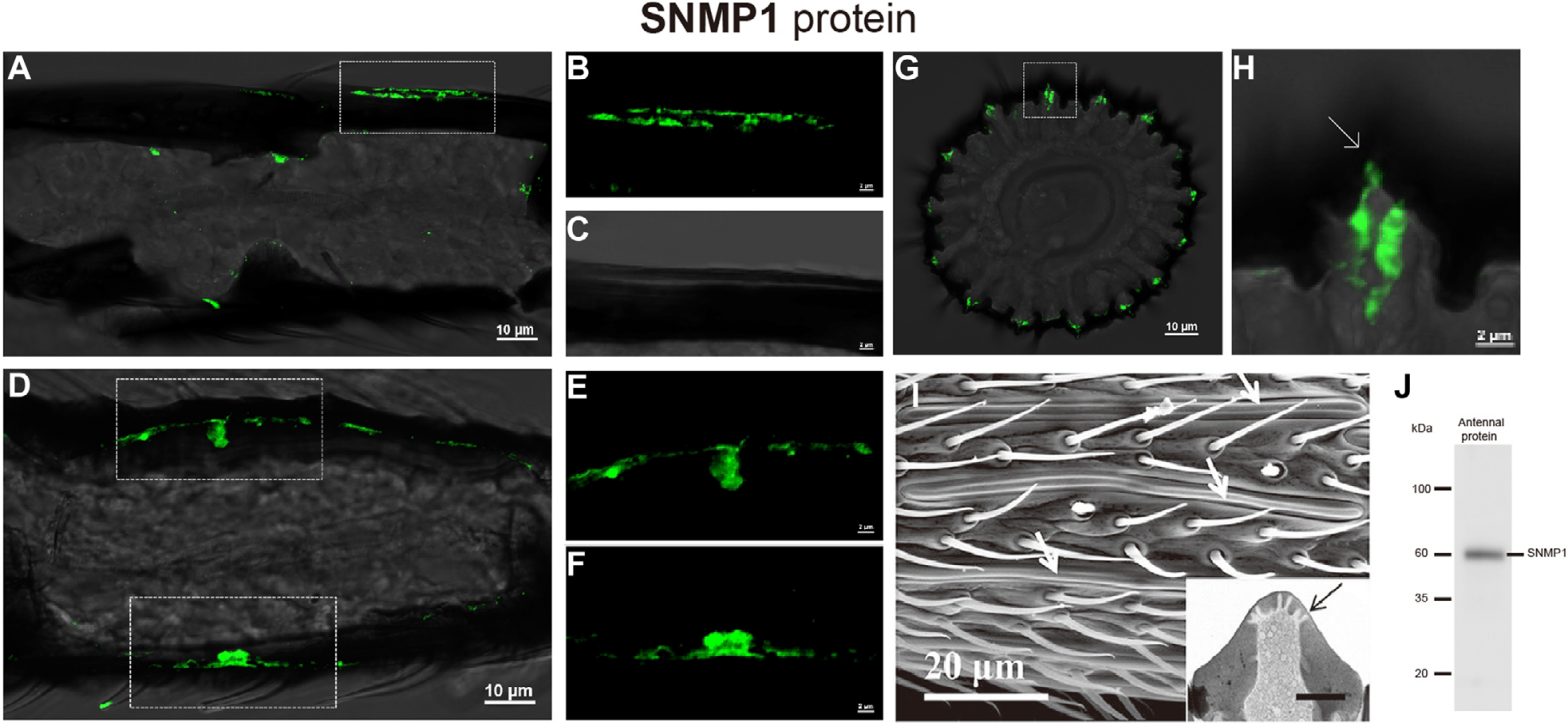
Localization of SNMP1 in antennae of male *Microplitis mediator*. MmedSNMP1 (green) is expressed in long axis (A–C), central dendritic bundle channel (D–F) and double ridges (G, H) of sensilla placodea. (I) Morphology of s. placodea (Wang *et al*., 2018). White arrows (H, I) indicate the morphology of s. placodea. Black arrow (I) indicates the transverse section of s. placodea where there are multiple pores on the wall. (J) Western blot analysis of the MmedSNMP1.

Fluorescence *in situ* hybridization (FISH) assay indicated that *MmedSNMP1* was expressed in cell clusters of male and female antennal flagella (Fig. 5). In Western blot analysis, staining of antennal extract with anti‐MmedSNMP1 antiserum showed a strong band at ∼60 kDa, which was a similar size to the predicted MmedSNMP1 (about 58.4 kDa) (Fig. 6J). The cellular localization clearly demonstrated that MmedSNMP1 was mainly expressed on the edge of the long axis (Fig. 6A–C) and double ridges (Fig. 6G, H) of s. placodea. Additionally, MmedSNMP1 was also found to be expressed in the central channel of s. placodea, over where dendrites of sensory neurons passed through (Fig. 6D–F).

**Figure 7:**
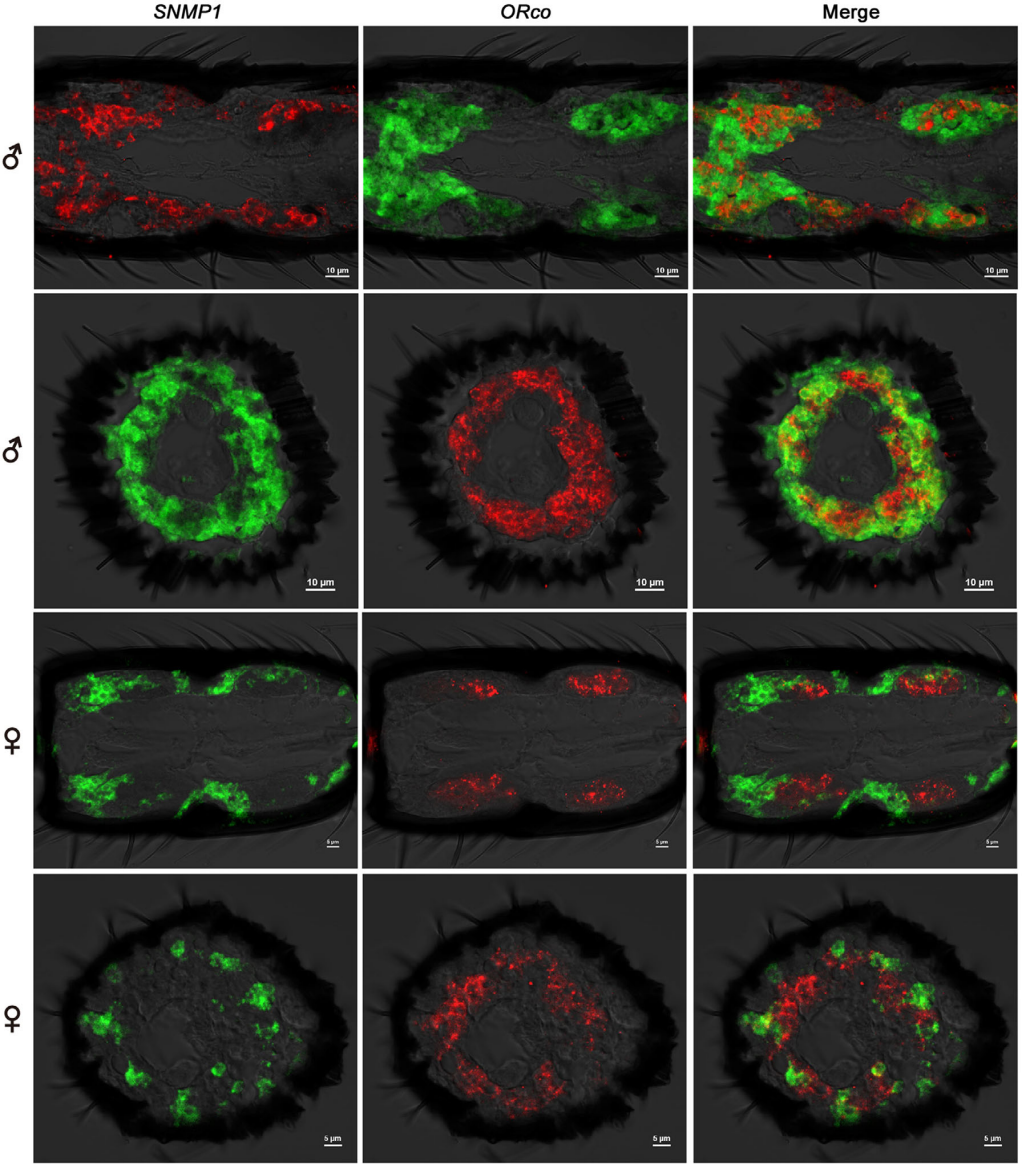
Co‐localization of *MmedSNMP1* and *MmedOrco* in antennae of *Microplitis mediator*. Combined DIG‐labeled and biotin‐labeled probes of *MmedSNMP1* and *MmedOrco* are hybridized to vertical and horizontal sections of antennae from both sexes. Left panel shows the *MmedSNMP1*, middle panel shows the *MmedOrco*, and right panel shows merged pictures.

**Figure 8:**
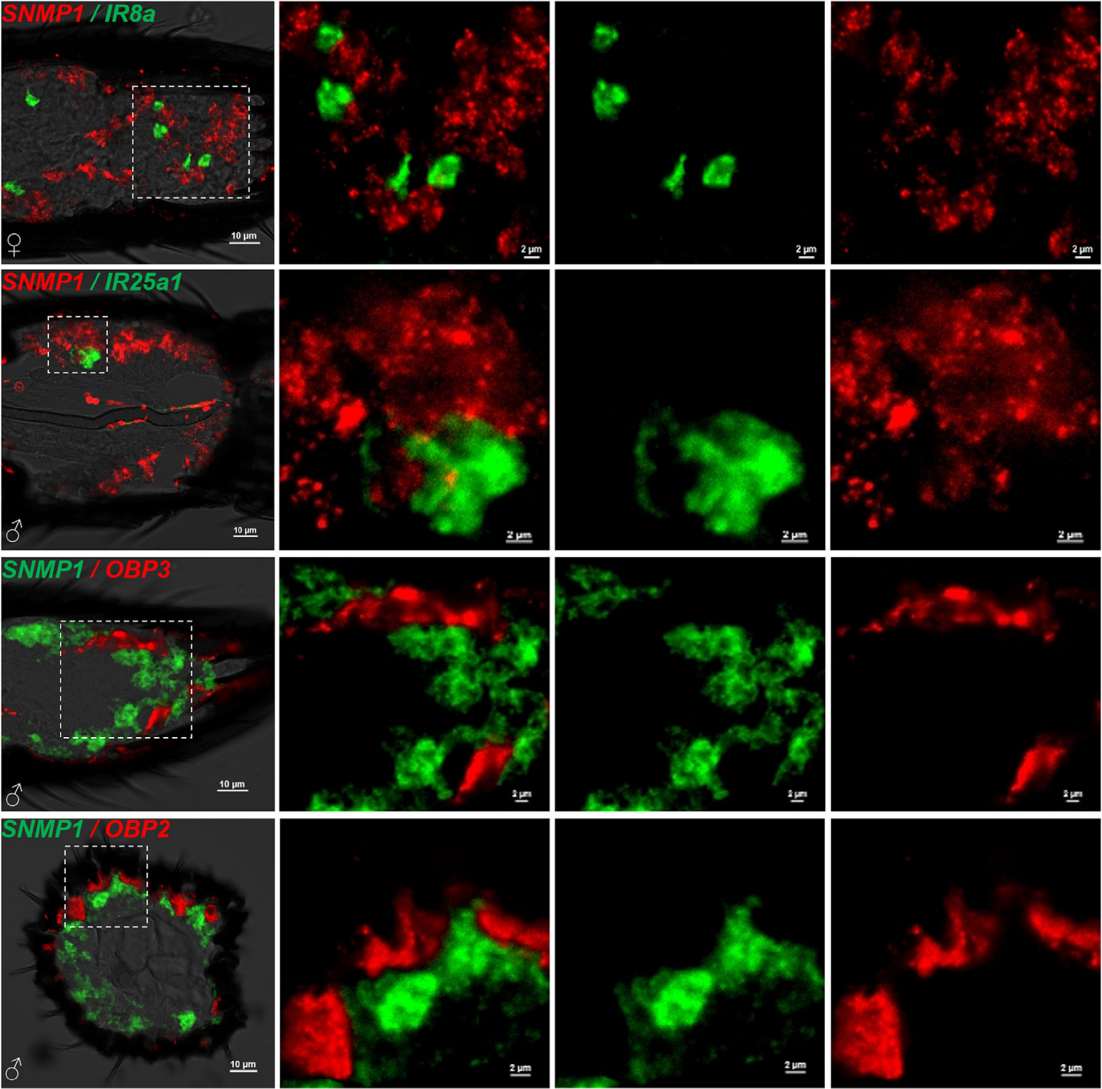
Double fluorescence *in situ* hybridization assays of *MmedSNMP1* with chemosensory genes in antennae of *Microplitis mediator*. DIG‐labeled and biotin‐labeled probes of *MmedSNMP1* and *MmedIR8a*/*IR25a1*/*OBP3*/*OBP2* are hybridized to vertical and horizontal sections of antennae. White dashed frame areas are illustrated at a higher magnification on the right.

To further investigate the expression and distribution characteristics of *MmedSNMP1*, other chemosensory genes including *MmedORco* (OR‐coreceptor), *MmedIR8a* and *MmedIR25a1* (IR‐coreceptors), *MmedOBP2* (expressed in olfactory sensilla) and *MmedOBP3* (expressed in gustatory sensilla) were selected to evaluate the co‐expression with *MmedSNMP1* in double FISH experiments. It was found that the expression levels of *MmedSNMP1* and *MmedORco* were significantly higher in male antennae segments than in females. The signals labeled by *MmedSNMP1* and *MmedORco* probe were partially overlapped on both longitudinal and horizontal sections of antennae in both sexes. Moreover, *MmedSNMP1*‐expressing cell clusters were closer to antennae cuticle than that of *MmedORco* (Fig. 7). However, there were no co‐expressions between *MmedSNMP1* and *MmedIR8a*, *MmedSNMP1* and *MmedIR25a*, *MmedSNMP1* and *MmedOBP2* as well as *MmedSNMP1* and *MmedOBP3*, respectively. Cells expressing *MmedOBP2* and *MmedOBP3* were obviously closer to antennae cuticles than that of *MmedSNMP1*, while compared with *MmedIR8a* and *MmedIR25a*, *MmedSNMP1*‐expressing cells were relatively clinging to antennae cuticles (Fig. 8).

## Discussion

In this study, two SNMP genes, *MmedSNMP1* and *MmedSNMP2*, were identified from antennae of *M. mediator*. The topo structure indicated that these two proteins contain two transmembrane domains and a large extracellular loop, which is similar to that of the vertebrate CD36 family (Rasmussen *et al*., 1998; Gruarin *et al*., 2000; Rogers *et al*., 2001a). The phylogenetic analysis indicated that MmedSNMP1 and MmedSNMP2 belong to two distinct subfamilies. High sequence similarities of SNMPs across Hymenoptera suggested their conserved roles in these species.

*MmedSNMP1* was mainly expressed in antennae of *M. mediator*, with higher expression level in male antennae than in females. The same cases were also reported in lepidopteran and dipteran insects, suggesting SNMP1 important roles in male chemoreception (Benton *et al*., 2007; Vogt *et al*., 2009; Gu *et al*., 2013; Liu *et al*., 2013; Liu *et al*., 2014; Zhang *et al*., 2015). In addition, our data demonstrated that expression levels of *MmedSNMP1* in both sexes were highly developmentally regulated. Similarly, *SNMP1* transcripts *of A. polyphemus*, *Manduca sexta* and *A. ipsilon* were sharply increased during 1–2 days prior to the adult eclosion or at the emergence day, which synchronized with the development of the insect olfactory system (Rogers *et al*., 1997, 2001a; Gu *et al*., 2013). SNMP1s were enriched in olfactory organs of mature insects indicating their important roles in chemosensory behavior of adult wasps and other species. In contrast, *MmedSNMP2* was widely expressed in various tissues of males, while being expressed relatively higher in female antennae than in other tissues. In *Cydia pomonella* (Huang *et al*., 2016), *Spodoptera exigua* (Liu *et al*., 2014) and *A. ipsilon* (Gu *et al*., 2013), *SNMP2s* are also broadly expressed in various tissues and relatively abundant in antennae of both sexes. Moreover, we found that the expression of *MmedSNMP2* was significantly increased at half‐pigmented pupae stage and kept a constant level at the subsequent developmental stages. Therefore, we speculated that SNMP2s may play multiple functions other than olfaction in wasps and other species.

There were multiple conserved intron insertion sites and phases of SNMP1s across distinct species, suggesting the evolutionary relatedness among these genes. In particular, SNMP1s from four hymenopteran species shared seven completely conserved intron insertion sites and phases, indicating their conserved roles across Hymenoptera insects. Simultaneously, similar sites are also present in other CD36 homologs of dipteran insects (Nichols & Vogt, 2008). The phenomenon of conserved intron insertion sites may be common characteristics of the SNMP/CD36 gene family.

Generally, SNMP1s are distributed in different types of sensilla. In *S. gregaria*, *S. litura*, *A. ipsilon*, *H. virescens* and *A. polyphemus*, SNMP1s are commonly expressed in pheromone‐sensitive sensilla (trichodea and basiconica) (Rogers *et al*., 1997, 2001a; Forstner *et al*., 2008; Gu *et al*., 2013; Zhang *et al*., 2015; Jiang *et al*., 2016). In *Drosophila*, SNMP1 mainly concentrated in the trichoid sensory cilia of antennae, is essential for detecting sex‐pheromone cVA (Benton *et al*., 2007; Jin *et al*., 2008). In addition, SNMP1 is co‐presented with pheromone receptor HR13 in the cells of sensory neurons in sensilla trichodea on antennae of *H. virescens*. Similarly, SNMP1 is also required for the activation of HR13 by lipid‐derived pheromone ligand (*Z*)‐11‐hexadecental (Benton *et al*., 2007; Große‐Wilde *et al*., 2007; Pregitzer *et al*., 2014). Our FISH results showed that *MmedSNMP1* was largely expressed in cell clusters of antennal flagella. Immunocytochemical localization further indicated that MmedSNMP1 was mainly expressed in the s. placodea of antennae. S. placodea of parasitoid wasp *M. croceipes* may be involved in the perception of plant‐emitted volatiles and sex pheromones (Ochieng *et al*., 2000; Bleeker *et al*., 2004; Baaren *et al*., 2007; Gao *et al*., 2007). In the previous study, s. placodea with wall pores were observed in antennae flagella of *M. mediator*, which are likely involved in olfactory perception of wasps (Wang *et al*., 2018). In short, MmedSNMP1 may also participate in both pheromones and general odors detection.

SNMP1s were commonly co‐expressed with pheromone receptors to detect specific sex pheromone components in some insects. In *Drosophila*, SNMP1 is required in pheromone detection of OSNs containing receptor OR67d (Benton *et al*., 2007; Jin *et al*., 2008). In *H. virescens*, SNMP1 co‐expressed with pheromone receptor HR13 or HR6 may be involved in the detection of corresponding sex‐pheromone components (Große‐Wilde *et al*., 2007; Pregitzer *et al*., 2014; Zielonka *et al*., 2018). Recently, it was suggested that BmorOrco, BmorSNMP1 and BmorOR1 formed a heteromer to detect sex pheromone bombykol in *B. mori* (Zhang *et al*., 2018). In the current study, double color FISH assays indicated that *MmedSNMP1*‐ and *MmedORco*‐expressing cells had higher numbers in males. Further analysis demonstrated that *MmedSNMP1* and *MmedORco* were expressed in partial overlapping OSNs in antennal flagella. So, there may be functional association between SNMP1 and ORs in pheromone detection of *M. mediator*.

The odorant binding protein LUSH is in addition to SNMP1 and OR67d essential for cVA detection in *Drosophila* (Xu *et al*., 2005; Benton *et al*., 2007; Jin *et al*., 2008; Laughlin *et al*., 2008). In *H. virescens*, pheromone‐binding proteins PBP1 and PBP2 are expressed in support cells around the pheromone receptors HR13 and HR6 expressing OSNs, whereas SNMP1 is co‐expressed respectively with the two receptors (Große‐Wilde *et al*., 2007; Zielonka *et al*., 2018). In our study, there was no co‐expression between *MmedSNMP1* and *MmedOBP2*, or *MmedSNMP1* and *MmedOBP3*. However, we could not exactly define the interaction between the SNMP1 and the selected MmedOBPs. Moreover, there was also no co‐expression between *MmedSNMP1* and *MmedIR8a*, or *MmedSNMP1* and *MmedIR25a1*, even though *MmedIR8*a was also expressed in s. placodea (Wang *et al*., 2016). We proposed that MmedSNMP1 and MmedIR8a are expressed in different OSNs locating in the same type of sensilla but perform distinct roles.

Overall, SNMP1 appears to play critical roles in chemoreception, especially in sex pheromone detection of *M. mediator*. In further studies, we will investigate the detailed roles of the SNMP family by using a *Xenopus* oocytes system and target gene knockout techniques. It will provide a basis for the development of pest control strategy by regulating chemical communication of natural enemies.

## Disclosure

The authors have declared that no competing interest exists.

## Supporting information


**Table S1**. Primers used in this study.
**Fig. S1**. Transmembrane domains and topological structures of MmedSNMP1 and MmedSNMP2.
**Fig. S2**. Sequence alignment of SNMP1 (A) and SNMP2 (B) from different hymenopteran species. Completely identical residues are marked in white letters with red background. Amino acids with physical and chemical properties are highlighted in red letters. The similar and identical residues are framed in blue rectangle.
**Supplemental material 1**. The amino acid sequences of SNMP used in phylogenetic tree analysis and sequence alignment.
**Supplemental material 2**. SNMP1 cDNA sequences used in alignment of intron insertion sites from different insect species: intron insertion sites (in cDNA sequences) are marked in yellow (the first nucleotide of an exon).
**Supplemental material 3**. The genomic sequence of *MmedSNMP1*.
