# Supplementary material for: Molecular characterization and expression of sensory neuron membrane proteins in the parasitoid Microplitis mediator (Hymenoptera: Braconidae)
Source: Insect Sci. 2019 Mar 14;27(3):425–39. doi: 10.1111/1744-7917.12667 (PMC7277062; doi:10.1111/1744-7917.12667)
Supplement: Supplementary file 1 — Table S1. Primers used in this study. Fig. S1. Transmembrane domains and topological structures of MmedSNMP1 and MmedSNMP2. Fig. S2. Sequence alignment of SNMP1 (A) and SNMP2 (B) from different hymenopteran species. Completely identical residues are marked in white letters with red background. Amino acids with physical and chemical properties are highlighted in red letters. The similar and identical residues are framed in blue rectangle. Supplemental material 1. The amino acid sequences of SNMP used in phylogenetic tree analysis and sequence alignment. Supplemental material 2. SNMP1 cDNA sequences used in alignment of intron insertion sites from different insect species: intron insertion sites (in cDNA sequences) are marked in yellow (the first nucleotide of an exon). Supplemental material 3. The genomic sequence of MmedSNMP1. [file INS-27-425-s001.docx]

**Table S1.** **Primers used in this study.**

| Primer Name | Sequence (5′-3′) |
| --- | --- |
| Full-length *SNMP* cloning | |
| SNMP1-F | ATGCTTTTATTCAAAAAACTTGGTATTG |
| SNMP1-R | TCAGGCTTCTACAGAGGGTGGA |
| SNMP2-F | ATGACTTCTTGTAGTACTCGATTTCG |
| SNMP2-R | TCAACTATTTATTGATAGAA |
| Gene structure |  |
| SNMP1-1-F | ACTTGGTATTGCCGGTGGTT |
| SNMP1-1-R | TGGCAAGAGAGCATTTGGGA |
| SNMP1-2-F | TCCCAAATGCTCTCTTGCCA |
| SNMP1-2-R | GCTTCTACAGAGGGTGGAGC |
| RT-qPCR | |
| SNMP1-F | TTAGAGGTAGGAATAGTTACGG |
| SNMP1-R | TAGTTGAATCAGTGCCATTG |
| SNMP2-F | AGACATATTATTTGACGGACTG |
| SNMP2-R | ATGAATAGACGCCTGGTT |
| β-actin-F | TGAGAAGATGACGCAGATTA |
| β-actin-R | ATCAAGAACGATACCAGTAGT |
| *In situ* hybridization |  |
| SNMP1-antisense-F | CTCGAGATGCTTTTATTCAAAAAACTTGGTATTGCCGG |
| SNMP1-antisense-R | TAATACGACTCACTATAGGGCGATCAGGCTTCTACAGAGGGTGGA |
| SNMP2-antisense-F | CTCGAGTATGCGACCCTGTCAAATAC |
| SNMP2-antisense-R | TAATACGACTCACTATAGGGAGACCACTGACCTTGTAGATATCT |
| ORCO-antisense-F | GGATCCATGATGAAAACAAAGCATCA |
| ORCO-antisense-R | TAATACGACTCACTATAGGGAGATTATTTAAGTTGTACTAATACC |
| IR8a-antisense-F | CGCGGATCCATGTCAAAAATCGGAATTAAGTTTATTG |
| IR8a-antisense-R | TAATACGACTCACTATAGGGAGATTAATCTTTATACGGAAAAGTTTTGGG |
| IR25a1-antisense-F | GAATTCGAGTACCGGATTTAGTTCTTGATACAAC |
| IR25a1-antisense-R | TAATACGACTCACTATAGGGAGATTCATCGCCAATCATAACTAAATCACAT |

F: forward primer; R: reverse primer.

**Fig. S1**


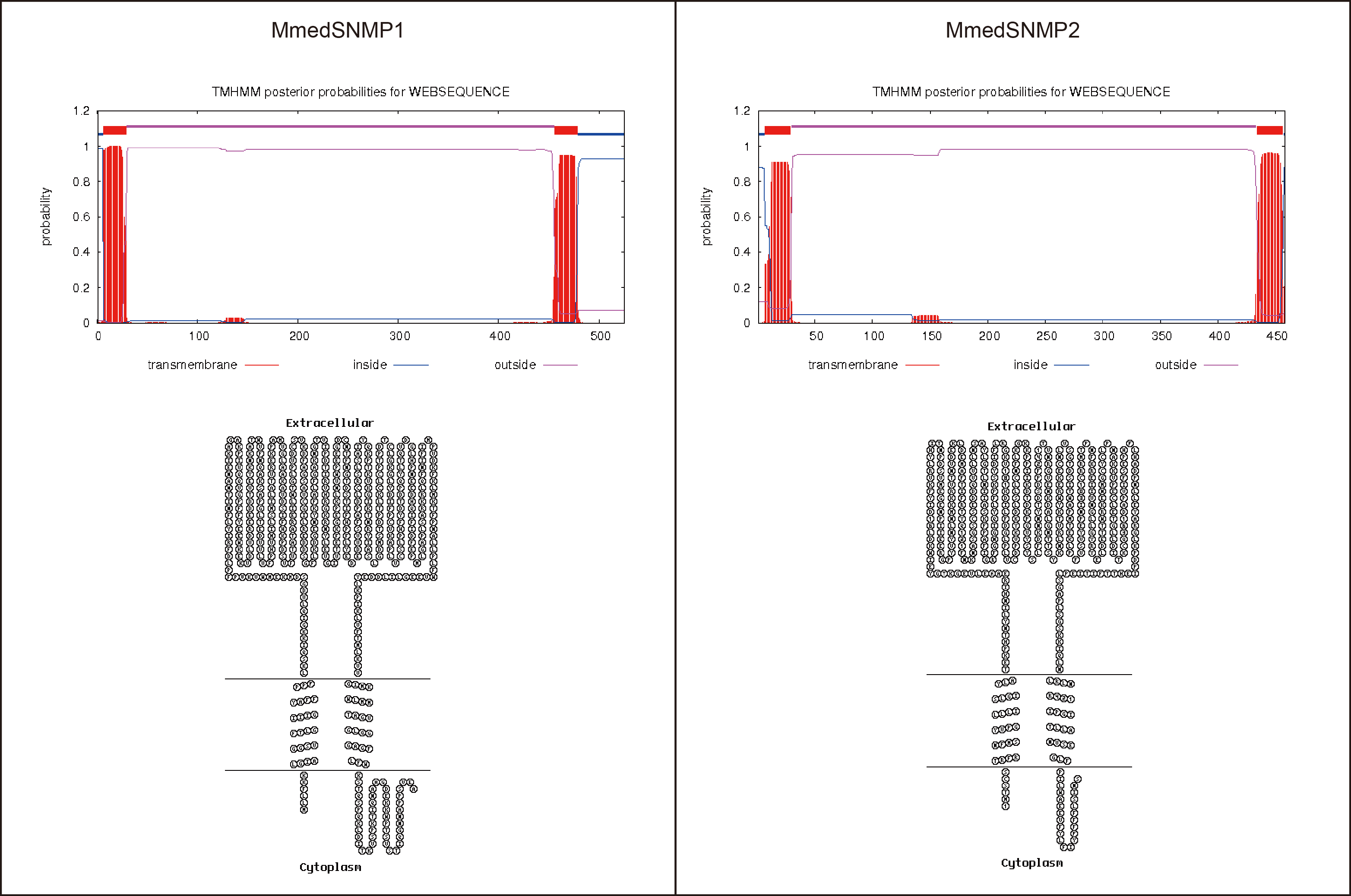


**Fig. S2**


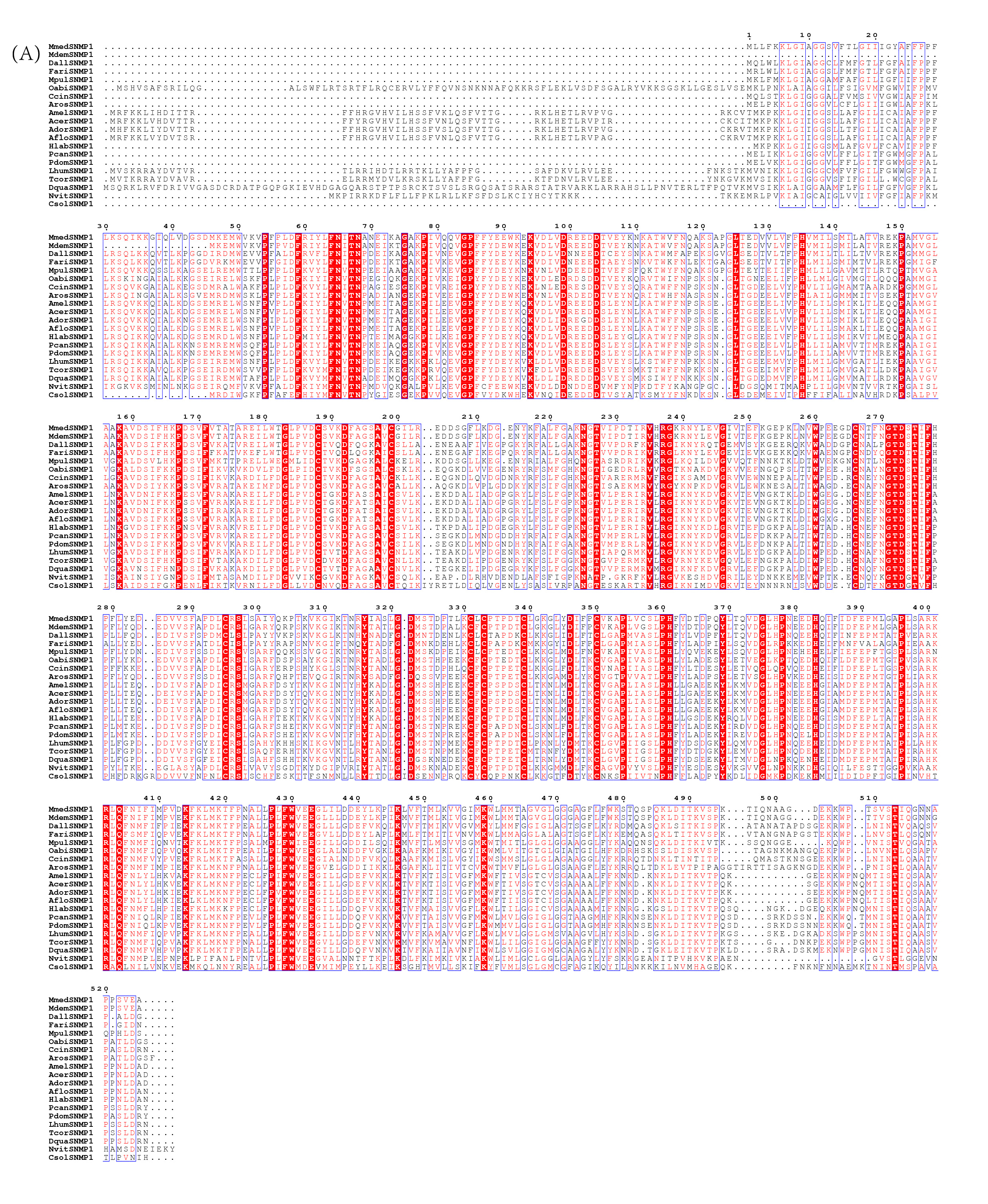


**Fig. S2**


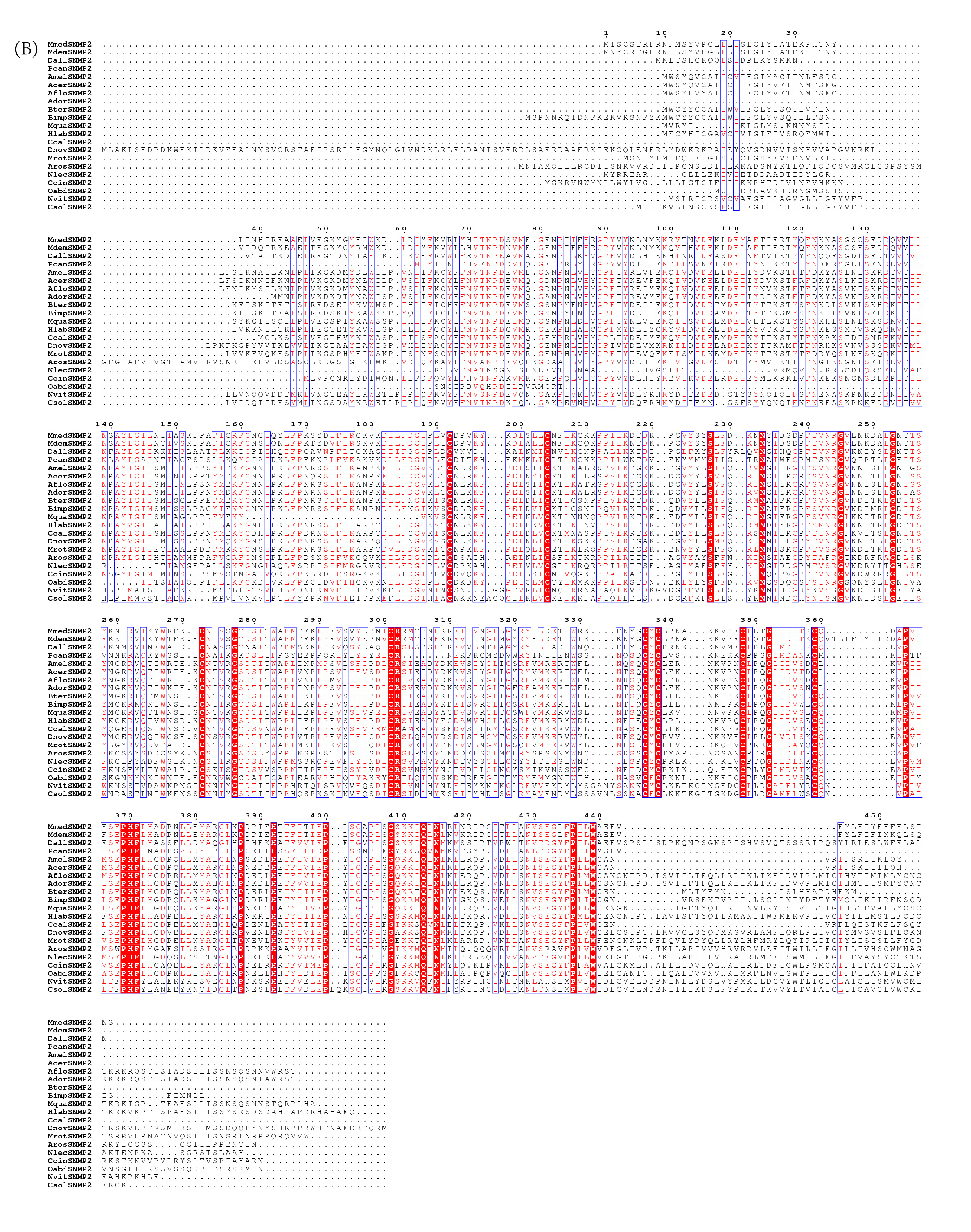


**Supplemental material 1**

The amino acid sequences of SNMP used in phylogenetic tree analysis and sequence alignment.

>MmedSNMP1 (gi|700275481|gb|AIU56857.1) [*Microplitis mediator*]

MLLFKKLGIAGGSVFTLGIIIGYAFFPPFLKSQIKKGIQLVDGSDMKEMWVKVPFPLDFRIYLFNITNANEIKAGAKPIVQQVGPFFYEEWKEKVDLVDREEDDTVEYKNKATWVFNQAKSAPGLTEDVVLVFPHVMILSMILATVREKPAMVGLAAKAVDSIFHKPDSVFVTATAREILWTGLPVDCSVKDFAGSAVCGILREDDSGFLKDGENYKFALFGAKNGTVIPDTIRVHRGKRNYLEVGIVTEFKGEPKLNVWPEEGDCNTFNGTDSTIFHPFLYEDEDVVSFAPDLCRSLSAIYQKPTKVKGIKTNRYIASLGDMSTDPTLKCLCPTPDTCLGKGLYDIFPCVKAPLVCSLPHFYDTDPQYLTQVDGLHPNEEDHQIFIDFEPMLGAPLSARKRLQFNIFIMPVDKFKLMKTFPNALLPLFWVEEGLILDDEYLKPIKLVFTMLKVVGIMKWLMMTAGVGLGGGAGFLFWKSTQSPQKLDITKVSPKTIQNAAGDEKKWPTSVSTIQGNNAPPSVEA

>MmedSNMP2 [*Microplitis mediator*]

MTSCSTRFRNFMSYVPGLLLISLGIYLATEKPHTNYLINHIREAAELVEGKYGYEIWKDLDIYFKVRLYHITNPDSVMEGENPIIEERGPYVYNLNMKKRVTNVDEKLDEMAFTIFRTYQFNKNASGSCSEDDQVVLLNSAYLGTLNIIASKFPAFIGRFGNGIQYLFPKSYDIFLRGKVKDILFDGLPLVCDPVKYKDLSLLCNFLKGKKPPIIKDTDKPGVYSYSLFDKNNYTDSDPFTVNRGVENKDALGNTTSYKNLRVTKYWREKECNLVSGTDSITWAPMTEKLPFVSVYEPNICRRMTPNFKREIIVNGLLGYRYELDETTWRKENMGCYCLPNAKKVPECLETGLLDITKCQDAPVIFSEPHFLHADPNLLEYARGLKPDPIEHTTFITIEPLSGAPLSGSKKIQLNLRLNRIPGITLLANVSEGLFPILWAEEVFYLFIYFFFFLSINS

>DmelSNMP1 (AAF55863, CG7000) [*Drosophila melanogaster*]

MQVPRVKLLMGSGAMFVFAIIYGWVIFPKILKFMISKQVTLKPGSDVRELWSNTPFPLHFYIYVFNVTNPDEVSEGAKPRLQEVGPFVFDEWKDKYDLEDDVVEDTVSFTMRNTFIFNPKESLPLTGEEEIILPHPIMLPGGISVQREKAAMMELVSKGLSIVFPDAKAFLKAKFMDLFFRGINVDCSSEEFSAKALCTVFYTGEIKQAKQVNQTHFLFSFMGQANHSDSGRFTVCRGVKNNKKLGKVVKFADEPEQDIWPDGECNTFVGTDSTVFAPGLKKEDGLWAFTPDLCRSLGAYYQHKSSYHGMPSMRYTLDLGDIRADEKLHCFCEDPEDLDTCPPPKGTMNLAACVGGPLMASMPHFYLGDPKLVADVDGLNPNEKDHAVYIDFELMSGTPFQAAKRLQFNLDMEPVEGIEPMKNLPKLILPMFWVEEGVQLNKTYTNLVKYTLFLGLKINSVLRWSLITFSLVGLMFSAYLFYHKSDSLDINSILKDNNKVDDVASTKEPLPSANPKQSSTVHPVQLPNTLIPGTNPATNPATHHKMEHRERY*

>DmelSNMP2 (EU189152, CG7422) [*Drosophila melanogaster*]

MIHWSLIVSALGVCVAVLGGYCGWILFPNMVHKKVEQSVVIQDGSEQFKRFVNLPQPLNFKVYIFNVTNSDRIQQGAIPIVEEIGPYVYKQFRQKKVKHFSRDGSKISYVQNVHFDFDAAASAPYTQDDRIVALNMHMNAFLQVFEREITDIFQGFANRLNSRLNQTPGVRVLKRLMERIRGKRKSVLQISENDPGLALLLVHLNANLKAVFNDPRSMSVSTSVREYLFDGVRFCINPQGIAKAICNQIKESGSKTIREKSDGSLAFSFFGHKNGSGHEVYEVHTGKGDPMRVLEIQKLDDSHNLQVWLNASSEGETSVCNQINGTDASAYPPFRQRGDSMYIFSADICRSVQLFYQTDIQYQGIPGYRYSIGENFINDIGPEHDNECFCVDKLANVIKRKNGCLYAGALDLTTCLDAPVILTLPHMLGASNEYRKMIRGLKPDAKKHQTFVDVQSLTGTPLQGGKRVQFNMFLKSINRIGITENLPTVLMPAIWVEEGIQLNGEMVAFFKKKLISTLKTLNIVHWATLCGGIGVAVACLIYYIYQRGRVVEPPVK*

>DpseSNMP1 (GA20018, confirmed to SNMP1Dmel model) [*Drosophila pseudoobscura*]

MKLDRMKLLFVSAGTLVFAILFGWVMFPKILKFMISKQVTLKPGTDVRELWSNTPFPLHFYFYVFNVTNPEDVSQGGRPRLQEVGPFVFDEWKDKIDLVDDVVEDSVTFTMRNTFIFNAEASYPLTGEETITLPHPIMQPGGITVQRERAAMMELIAKAMSLVFPGAKAFLSAPFMDLFFRGIDVDCSPDDFAAKALCTVFYTGEVKQAKQVNQTHFLFSFMGQANHSDAGRFTVCRGVKNNKKLGKVIRFAEETEMDVWPGDECNQFEGTDSTVFPPGLKKEEGLWAFTPDLCRSLGATYVRKSSYHGMPSTRYTLDLGDMRSEEKLHCFCDDPEDLDTCPPRGTMNLAPCVGGPLLASMPHFYNGDPKLVAAVDGLHPNEKDHAVYIDFELMSGTPFQAAKRLQFNLDMEPVEGIEALKNLPKLILPLFWIEEGVHLNKTYTNMVKYTLFLGLKFNSGLRWTLITLSLVGLMSAAYLFYQNSDSLDITLPPKILKEVNKVADAAMNSKMFPEKAPTTPQTTIPGTNPPTNHGAQPPPAVASVPGIIPPLSLKMEQAQRY*

>DpseSNMP2 (corrected to Dmel model 7/25/08) (GA20338, scaffold: CH379070) [*Drosophila pseudoobscura*]

MLHWSLIVSALGVCVAVLGGYCGWSLFPNMVHKKVEQSVILADGSEQYKRFVNLPQPLNFKVYIFNVTNPDMIQHGAIPIVEEIGPYVYKQYRHKKVKHFSRDGSKITYVQNVHFDFDADASAPYTQDDRIVALNMHMNAFLQVFEREITDIFQGFANRLNSRLNRTPGVRILKRLMERIRGKRKSVLQISENDPGLALLLVHLNANLKAVFNDPRSMFVHTSVREYLFDGVRFCINPQGIAKAICNQIKESGSKTIREQSDGSLAFSFFGHKNGSGHDVYEVHTGKGDPMKVLEIQKLDDSHNLQVWLNASTEGETSVCNQINGTDASSYPPFRQRGDSMYIFSADICRSVQLFYQADIQYEGIPGYRYSIGENFINDIGPEHDNECFCVDKLANVIKRKNGCLYAGALDLTTCLDAPVILTLPHMLGASNEYRKMIRGLNPDAKKHQTFVDVQSLTGTPLRGGKRVQFNMFLKSINRIGITENLTTVLMPAIWVEEGIQLNGEMVAFFKKKLINTLKALNIVHWAALCGGAGVALISLLYYLYQKGRGEEAPLK*

>AaegSNMP1 (EU246941, FJ387158) [*Aedes aegypti*]

MLIKNRKNLMLKPGTQMRGMFEKIPFPLDFKLYLFHVTNPDVVMKGGKPHVREIGPYFFEEWKEKYDTVDNEEDDTLTFTLKNTWIFRPDLTKPLTGDEMITIPHPLILGALLMVQRDREAMMPLVSKGMDIIMNPLTTGFLTTRVMDLLFDGILIDCSSQEFSAKALCSGLESEGAVMPFNETHFKFSMFGLKNGTDAGRWVVYRGVKNIMDLGRVVSFNDETEMDIYDGDECNRYIGTDSTIFPPFLTTKDKLWAWSPEICRSIGAEYGGKSKYAGLPMSFFKLDFGDARNEPEHHCFCRDPPDICPPKGTIDLAPCLGAPIIGSKPHFYDSDPKLLAAVDGLTPNEKDHDVYIHFQLLSGTPVSAAKRLMFSMEIEPIRDHAVLGNLPTVILPLFWAEEGASLNKTWTNQLKYTLFLGLRFNTAVKWLTIIIGTIGTIVGGFMHYKRTTKTVNVTPVQSVNGSSAKGNGAGMTVVGHQPDSKGGSVTTPVIPSAKDLLQNSRNLPTVIEGLDRPQKVTVTEMQERY*

>AaegSNMP2 (EU189151, FJ387159) [*Aedes aegypti*]

MMVMNTELRQDTPQFKRWEAVPQPLDFKVYIFNVTNPYEVQMGRRPRVVEVGPYVYFQYCHKDNIRFSRDRSKVHFSQQQMYVFDAESSYPLTENDQLTVLNMHMNSILQIIDTQAKETITNFRSDVNNTLEKIPVVRVIKRIIEKTTPIQSILQLAEDETYDSLRLINAELNRIFGRPDSMFLRTTPREFLFEGVPFCVNVIGIAKAICKEIEKRNTKTIRVQPDGSMKFSFFNHKNMTNDGTYTINTGIKEPALTQMIEYWNGRNTLDRWINQSAGSSSKCNKIVGTDGSGYPPFREGVERM

TIFSSDICRTVDIKYVGPSSYEGIPALRFETDSHFLNEIGPEYGNDCYCVNRIPKAIVKNNGCLYKGALDLSTCFDAPVVLTHPHMMGAAQEYTSLIDGLYPDPEKHQIFVDVEPLTGTPLNGGKRVQFNMFLRRIDSIRLTDRLQTTLFPVLWIEEGIALNEDMVKLIDDSLMKVLTLLDIVQWVMIGSGLLLAIIMPIVYFIKRKPSSGSITPTLTTTTSTVSISDGGGLGGNPQK*

>AgamSNMP1 (EAA07966 + EAA07986, corrected to SNMP1Aaeg model) [*Anopheles gambiae*]

MELKERNFKKIGLICVAVLLCGMVFSYGIFPSILRFMIKQNVLLKPGTQIRDMFEKIPFPLDFKLHIFNVTNPDEIMRGGKPRVNDIGPLYFEEWKEKYDTVDNVEEDTLTFTLRNTWIFRPDLSALTGEEIVTIPHPLIMGVLLMVQRDREAMMPLVKKGVNILFDPLESAFLKVRIMDLLFDGIYVDCSSQDFAAKALCSGMDSEGAVMPHNETHYKFSFFGMRNHTEAGRWVVYRGVKNIRDLGRVVSYNEETEMDIWDGDECNQYIGTDSTIFPPFLTAQDRLWAWSPEICRSLGAHYVH KSKYAGLPMSYFELDFGDLKNEPHNHCFCRDAPDDCPPKGTMDLSPCLGGPIIGSKPHFYGADPKLVEAVDGLAPNKAAHDVYIHFELASICWVSPVSAAKRLQFSMELGPIRDHELFGQLPDVILPMFWAEEGASLNKTWTNQLKYQLFLGLKFNATVKWLTIIIGTVGAVGSAYMYFRKETKTTDVAPVDVSTPDTNPSSAKDGVVNVSLGRNLPPVIDGLDKPPK

LRATELQQERY*

>AgamSNMP2 (EAA11629, corrected to SNMP2Aaeg model) [*Anopheles gambiae*]

ATELRQGTDQYKRWEALPQPLDFKVYIFNVTNPYEVMQGRRPKVVEVGPYVYFQYRQKDNVRFSRDRSKVHFSQQQMYVFDAESSYPLTENDELTVLNMHMNSILQIIDNQAKETITNFRSDVNNTLEKIPVVRVIKRIIERTTPIQSILQIAEDETYDSLRLINVELNRIFGRPDTMFLRTTPKQFLFDGVPFCVNVIGIAKAICKEIEKRNTKTIRTMPDGSLRFSFFSHKNMTDDGMFTINTGIKDPSRTQMIELWNGRTTLDVWNNRSSGLSSSCNKIHGTDGSGYPPFRTGVERMTIFSTDICRTVDIKLTGSSSYEGIPALRYEIDNNFLHEIGPEYGNDCYCVNKIPKSIVKSNGCLYKGALDLSNCFDAPVVLTLPHMLGVAEEYTALIDGMDPEPERHQIFVDVEPYTGTPLNGGKRVQFNMFLRRIDAIKLTDRLQPTLFPVIWIDEGIALNEDMVKLIDDSLMKVLSLLDVVQWVLIGVGLLLAVLMPTVYFVKRCRGEGSRTVSPAVTATTSAASLSTVAGVTGDRSK

>BmorSNMP1 (cDNA: AJ251958) [*Bombyx mori*]

MQLAKPLKYAAISGIVAFVGLMFGWVIFPAILKSQLKKEMALSKKTDVRKMWEKIPFALDFKIYLFNYTNAEDVQKGAVPIVKEVGPFYFEEWKEKVEVEENEGNDTINYKKIDVFLFKPELSGPGLTGEEVIVMPNIFMMAMALTVYREKPAMLNVAAKAINGIFDSPSDVFMRVKALDILFRGIIINCDRTEFAPKAACTTIKKEAPNGIVFEPNNQLRFSLFGVRNNSVDPHVVTVKRGVQNVMDVGRVVAIDGKTKMNVWRDSCNEYQGTDGTVFPPFLTHKDRLQSFSGDLCRSFKPWFQKKTSYNGIKTNRYVANIGDFANDPELQCYCDSPDKCPPKGLMDLYKCIKAPMFVSMPHYLEGDPELLKNVKGLNPNAKEHGIEIDFEPISGTPMVAKQRIQFNIQLLKSEKMDLLKDLPGTIVPLFWIEEGLSLNKTFVKMLKSQLFIPKRVVSVVCWCMISFGSLGVIAAVIFHFKGDIMHLAVAGDNSVSKIKPENDENKEVGVMGQNQEPAKVM*

>BmorSNMP2 [*Bombyx mori*]

MLAKYTKTIFSVSVAFLVVSIVLATWGFPKIIRKQIQKNVQISNTSKMYDKWVKLPMPLDFKIYVFNVTNRDAINQGEKPNLKEIGPYVYKQYREKIILGYGDNDTIKYNLKKTFVFDPVASGDLREDDELTVINFSYMAAIISVQEMMPAAVGMINRALEQFFTNLTDPFQTVKVKDLFFDGLFLNCEGDNTALGLICGKIRAEKPPTMRISKSANGFYFSMFSHMNRTVSGPYEMVRGTENLSDLGHVISYQGKRIMSAWDDQYCGQLNGTDSTIFPPLEDGNIPEKLYTFEPDICRSLFAS

LVGKDTLFNISTYYYEISDMTLGSKSANPDNKCFCKrngsvkhdgcllmgvlnlapcqgAPAIASLPHFYLGSDELADFFGDGIKPDKEKHNTYVHLDPITGVVIKGVKRLQFNIELRNVPSVPQLKEVPSGLFPLLWIEEGAEIPEWLRKEIMDSHTMLWYVDAARWLVLAVAVVAVLVSATLVARSAALIPWPRNSNSISFILGNSVNTSKVHS*

>ApolSNMP1 (AAC47540.1) [*Antheraea polyphemus*]

MLLPKPLKYAAIGGGVFVFGILIGWVIFPVILKSQIKKEMALSKKTDLRQMWEKVPFALDFKVYIFNYTNVDEIQKGAKPIVKEIGPYYFEEWKEKVEVEDHEENDTITYKRLDVFHFRPDLSGPGLTGEEVIIMPHLFILAMVATINREKPSMLNVVEKSINGIFDNPKDVFLRVKAMDIMFRGIIINCDRTEFAPKAACTKMKKDAVTGVIYEPNNQFRFSLFGTRNNTVNPDVVTVKRGIKNIMDVGQVVALNGKPQIDIWRDHCNEFQGTDGTVFPPFLTYKDRLQSFSFDLCRSFKAWFQKKTSYKGIKTNRYIANVGDFANDPELQCFCDTPDECLPKGIMDIRKCLKVPMYVSLPHFLETDTSVTNQVKGLTPDPNEHGIIADFEPLSGTLMDAKQRMQYNIKLLRTDKIAIFKDLPDSIVPCFWVHEGILLNKTFVKMLKHQLFIPKRIVGVIRWWMVSFGLIAVLAGVMYHFKDNIMGWAAKGESTTAKVNPEDGSNEQRGVSVIGQDREPPKVTM

>ApolSNMP2 (CAP19029.1) [*Antheraea polyphemus*]

SMMYDKWLKLPMPIDFKVYVFNVTNPDDVNNGAKPILKELGPYVYRQYRERTVLGYGANDTIRYMLKKNFVFDPEASNGVTEDDEIVVINFAYMGAILTIHDIMPGATGMLNRALEQFFPNLTDPFHRVKVRDLFFDGTFLNCDGDNAALTLVCAKIKMDSPQTMRPAEDGVNGFYFSMFSHMNRTESGPYEMVRGRENVYELGNIISYMGKNSTGVWGDRYCDAINGSDSTIYPPIDVNNVPERLYSFEAEVCRSLYVSFVGKRTLFNITTYYYEIPESALAAKSANPNNKCFCKKNWSANHDGCLLMGIFSLMACQGAPAIASLPHFYLGSEELLEFFDGGIMPDKEKHQSFVHIDPVSGAVIKGLKRLQFNIELRNIPNIPQLQDVPTGLFPLLWVEEGAEITKSIGQELRDAHTLLSYVEVARWLILGIALLVALVAAVVLARSTSLIAWPRNSNSVSFILGPGINNVNKVRQ

>TcasSNMP1 (XP_001816436.2) PREDICTED [*Tribolium castaneum*]

MKPIRRKDFLFLLFPKLRLLKFSFDSLKCIYHCYTKKKTKKEMRLPVKIAIGCAIGLVVIIVFGFIAFPKMIKGKVKSMINLNKGSEIRQMFVKVPFALDFKIYMFNVTNPMDVQKGALPVLKEVGPFCFEEWKEKVDLDDNDDEDVMFYNPKDTFYKANGPGCLDGSQMITMAHPLILGMVNTVVRTKPGAISLISKAINSIYGNPDSIFMTASAMDILFDGVVIKCGVKDFAGKAVCSQLKEAPDLRHVDENDLAFSFIGPKNATPGKRFKVLRGVKESHDVGRILEYDNKKEMEVWPTKECNQYKGTDGTVFPPYLTKEEGLASYAPDLCRSLVAVYSGDTKYDGIPVRIYTATLGDMSKNADEKCYCPTPDTCLKKGMMDLFKCAGVPVYVSLPHFYESDESYVKGVVGLNPNKKDHGIQILFESTTGGPVKAAKRLQFNMPLEPNPKLPIFANLPNTVLPLFWVEEGVALNNTFTKPLKDLFKIMKIVKIAKWLIMLGCLGGLGAAGYLYFSKKGEANITPVHKVKPAENGVSTLGGEVNHAMSDNEIEKY

>TcasSNMP2 (XP_970008.1) PREDICTED [*Tribolium castaneum*]

MGCSCCTIKVLLVCVVISVALLIVSLALAFKVFPDLLESEVNKAVRLEDGTKQYDRFVELPFPVDFKVYLFNVSNPQQVLDGTEKPKLEEIGPFVYKQYRKKTILGKNEEEDTISYTQKETFEFDAEASKPLTEESVVTVLNPALMSIYQLAEDLHLAGAADTCIKQTFENNQGKVFIEANVRKLLFDGFSFCKNTSPGICGLVNDLICAIAATKRNSDLVLPDYSLIFSYLNYKRKPDDGKYTVKRGLTNIEKLGHIVAWNDSLYTKFWGEGTTCSEVKGTDSTLYPPRVTTDSAFYIYSTDICRFVKINYKGEESYKGIDGYLFETSEDTLRSSAPEEDCYCSKLSRDMEGKKSCFLDGVIDMQTCFGVPVLFSFPHFLWADNKYLSAVEGLNPVEEKHKTYLVVEPNTGTPLKGMKRIQLNGVIRPIVGIKSMLQTKRALLPLLWIEEGVSLPQKYVDELKSSYFDKVQIVDGVRYALIVISAILVGAFGIIILRKRSHAKHHV

>AmelSNMP1 (XP_006562934.1) [*Apis mellifera*]

MRFKKLIHDITTRFFHRGVHVILHSSFVKLQSFVTTGRKLHETLRVPVGRKCVTMKPKKLGIIGGSLLAFGILICAIAFPPFLRSQVKKQIALKDGSEMRELWSNFPVPLDFKIYLFNVTNPMEITAGEKPILEEVGPFFYDEYKQKVDLVDREEDDSLEYNLKATWFFNPSRSEGLTGEEELIVPHVLILSMIKLTLEQQPAAMGILNKAVDNIFKKPESVFVRAKAREILFDGLPVDCTGKDFASSAICSVLKEKDDALIADGPGRYLFSLFGPKNGTVLPERIRVLRGIKNYKDVGKVTEVNGKTKLDIWGEGDCNEFNGTDSTIFAPLLTEQDDIVSFAPDICRSMGARFDSYTKVKGINTYHYKADLGDMSSHPEEKCFCPSPDSCLTKNLMDLTKCVGAPLIASLPHLLGAEEKYLKMVDGLHPNEEEHGIAMDFEPMTATPLSAHKRLQFNLYLHKVAKFKLMKNFPECLFPIFWVEEGILLG

DEFVKKLKTVFKTISIVGFMKWFTIVSGTCVSGAAAALFFKNKDKNKLDITKVTPQKGEEKKWPNQMTISTIQSAAVPPNLDAD

>AmelSNMP2 (XP_016767241.1) [*Apis mellifera*]

MWSYQVCAIICVIFGIYACITNLFSDGLFSIKNAILKNLPLIKGKDMYDEWILPVNLIFKCYFFNVTNPDEVMEGNNPNLVEYGPFTYREVFEKQIVDVDEELDEIIYDVKSTFTFDKYASLNISKRDTVTILNPAYIGTISMLTTLPPSYIEKFGNNIPKLFPNRSSIFLKANPKEILFDGVKLTCNERKFPELSTICKTLKALRSPVLKEGEKEGVYYLSIFQRVNGTIRGRFSVNRGVNNISELGNIGSYNGRRVQTIWRTEKCNTVRGSDTITWAPLINPMPSVLSFIPDLCRSIEADYDKEVSIYGLIGSRFVMRERTWFLNQSQCYCLERNKVPNCLPQGLIDVSDCLKVPIIMSEPHFLHGDPQLLMYALGLNPSEDLHETFIVIEPYTGTPLSGQKKIQLNLKLERQPVDLLSNISEGYFPLLWCANVRIFSKIIKLQY

>AfloSNMP1 (XP_012339100.1) PREDICTED: LOW QUALITY PROTEIN: sensory neuron membrane protein 1-like [*Apis florea*]

MRFKKLVYDVTSRFFHRGVHVILHSSFVNLQSFVTTGRKLHETLRVPAGCKRVTMKPKKLGIIGGSLLAFGILICAIAFPPFLKSQVKKQIALKDGSEMRELWSNFPVPLDFKIYLFNVTNPMEITAGEKPILEEVGPFFYDEYKQKVDLVDREEDDSLEYNLKATWYFNPSRSEGLTGEEELVVPHVLILSMAKLTLEQQPAAMGILNKAVDSIFKKPSSVFVRAKAREILFDGLPVDCTGKDFATSAICSVLKEKDDALIADGPGRYLFSLFGPKNGTVLPERIRVLRGIKNYKDVGKVTEVNGKTKLDIWGXGDCNEFNGTDSTIFAPLLTEQDDIVSFAPDICRSMGARFDSYTQVKGINTYHYKADLGDMSSHPEEKCFCPTPESCLTKNLMDLTKCVGAPLIASLPHLLGAEEKYLKMVDGLHPNEEEHGIAMDFEPMTATPLSAHKRLQFNLYLHKIEKLKLMKNFPECLFPVFWVEEGILLGDEFVKKLKTVFKTISIVGFMKWFTIISGTCISGAAAALFFKNKDKNKLDITKVTPQKGEEKKWPNQLTISTIQSAAVPPNLDAN

>AfloSNMP2 (XP_003694668.1) PREDICTED: sensory neuron membrane protein 2-like [*Apis florea*]

MWSYHVYAIICLIFGIYVFTTNMFSEGLFNIKYSILKNLPLVKGKDMYNAWILPVSLIFKCYFFNVTNPDEVMQGDNPNLVEYGPFTYREIYEKQIVDVDEELDEIIYNVKSTFTFDKYASVNISKHDTVTILNPAYIGTISMLTNLPSNYMEKFGNNIPKLFPNRSSIFLKANPKEILFDGVKLTCNERKFPELSTICKTLKATRSPVLKQGEKDGVYYLSVFQRINGTIRGRFSVNRGVNNISELGNISSYNGKRVQTIWRTEKCNTVRGSDTITWPPLVNPLPMVLTFIPDLCRTVEADYDKEVSIYGLIGSRFVMKERTWFMNRSQCYCLERNKVPNCLPQGLIDVSDCLKVPIIMSEPHFLHGDPRLLMYARGLNPDEDLHETFIVIEPYTGTPLSGQKKIQLNLKLERQPVDLLSNISEGYFPLMWCANGNTPDLSVIILTFQLLRLIKLIKFLDVIPLMIGIHVTIMTMLYCNCTKRKRQSTISIADSLLISSNSQSNNVWRST

>NvitSNMP1 (XP_001606602.1) PREDICTED: sensory neuron membrane protein 1-like [*Nasonia vitripennis*]

MKPIRRKDFLFLLFPKLRLLKFSFDSLKCIYHCYTKKKTKKEMRLPVKIAIGCAIGLVVIIVFGFIAFPKMIKGKVKSMINLNKGSEIRQMFVKVPFALDFKIYMFNVTNPMDVQKGALPVLKEVGPFCFEEWKEKVDLDDNDDEDVMFYNPKDTFYKANGPGCLDGSQMITMAHPLILGMVNTVVRTKPGAISLISKAINSIYGNPDSIFMTASAMDILFDGVVIKCGVKDFAGKAVCSQLKEAPDLRHVDENDLAFSFIGPKNATPGKRFKVLRGVKESHDVGRILEYDNKKEMEVWPTKECNQYKGTDGTVFPPYLTKEEGLASYAPDLCRSLVAVYSGDTKYDGIPVRIYTATLGDMSKNADEKCYCPTPDTCLKKGMMDLFKCAGVPVYVSLPHFYESDESYVKGVVGLNPNKKDHGIQILFESTTGGPVKAAKRLQFNMPLEPNPKLPIFANLPNTVLPLFWVEEGVALNNTFTKPLKDLFKIMKIVKIAKWLIMLGCLGGLGAAGYLYFSKKGEANITPVHKVKPAENGVSTLGGEVNHAMSDNEIEKY

>NvitSNMP2 (XP_016840413.1) PREDICTED: sensory neuron membrane protein 2 [*Nasonia vitripennis*]

MSLRICRSVCVAFGFILAGVGLLLGFYVFPLLVNQQVDDTMKLVNGTEAYERWETLPIPLQFKVYFFNVSNPDEVQNGAKPIVKEVGPYVYDEYRHKYDITEDEDGTYSYNQTQLFSFNENASKPNKEDDNIIVAHLPLMAISLIAEKRLMSELLGTVVPHLFDNPKNVFLTTTVKKFLFDGVNINCSNGGGTVRLICNQIRRNAPAQLKVPDKGVDGPFVFSLLSYKNNTHDGRYKVSSGVKDISTLGEIYAWKNSSTVDAWKPNGTCNNIYGTDTTIFPPHRTQLSRVNVFQSDICRTVNLHYNDETEYKNIKGLRFVVEKDMLMSGANYSANKCYCLKETKGINGEDGCLLDGALELYRCQNVPLVLTFPHFYLAHEKYRESVEGLNPDKSKHEIFVELEPKSGTVLRGSKRVQFNIFYRPIHGINLTNKLAHSLMPVFWIDEGVELDDPNINLLYDSLVYPMKILDGVYWTLIGLGLAIGLISMVWCMLFAHKPKHLF

>CsolSNMP1 (XP_011499316.1) PREDICTED: sensory neuron membrane protein 1-like [*Ceratosolen solmsi marchali*]

MRDIWGKFPFAFEFHIYMFNVTNPYGIESGEKPVVQEVGPFVYDKWHEKVNQIDEEDDDTVSYATKSMYYFNKDKSNGLSDEMEIVIPHFFIFALINAVHRDKPSALPVLSKAIDSIFGKPENLFIKTKVRNILFDGLLVDCNVQDFAGSAVCTQIKIYRETLDIQLVGENLYSASIVRPANGTESKARTRVHRGIKNIMDVGKVIEYNNNRNISVWDDEYCDTFNGTDGTVFHPHFDRKGRDDVVVFNPNLCRSISCHFESKTTFSNMNLLRYTTDLGIDSENNPRQKCYCQPPNKCLKKGTFDTYKCNKSPIIVTNPHFFLADPYYKDLIDGMKPDKEKHMIIIDIDPFTGIPINVHTRAQLNILVNKVEKMKQLNNYREALLPIFWMDEVMIMPEYLLKEIKSGHTMVLLSKIFKYFVMLSGLGMCGFAGIKQYILRNKKKILNVMHAGEQKFNKNFNNAEMKTNINTMSPAVATLPVNIH

>CsolSNMP2 (XP_011505789.1) PREDICTED: sensory neuron membrane protein 2 [*Ceratosolen solmsi marchali*]

MLLIKVLLNSCKSLSIIFGIILTIIGLLLGFYVFPLVIDQTIDESVMLINGSDAYKRWETLPIPLQFKVYFFNVTNPDKIQLGAKPEVNEVGPYIYDQFRHKYDIIEYNGSFSYYQNQIFKFNEEASKPNKEDDVITVVHLPLMMVSTIAENRMPVFVNKVIPTLFYEPKNVFIETTPKEFLFDGIHIACNKKNEAGQGILKLVCKEIKKFAPIQLEELSDGRFKFSLLSYKNNTNDGHYNISNGVKNIDSLGEILSWNDASTLNIWKFNSSCNNIYGSDTTIFPPHQSPKSKIKVFQSDICRSIDLHYKSEIIYHDISGLRYAVENDMLSSSVNLSSNACFCLNKTKGITGKDGCLLDGAMELWSCQNVPAILTFPHFYLANEEYKNTIDGLTPNESLHLTFVDLEPLQKSGIVLRGSKRVQFNIFYRIINGIDITKNLTNSLMPIVWIDEGVELNDENIILIKDSLFYPIKITKVVYLTVIALGLTIC

AVGLVWCKIFRCK

>MsexSNMP1 (AAG49366.1) sensory neuron membrane protein 1 [*Manduca sexta*]

MRLARGIKYAVIGAGVALFGVLFGWVMFPAILKSQLKKEMALSKKTDVRKMWEKIPFALDFKIYLFNYTNPEEVQKGAAPIVKEVGPYYFEEWKEKVEIEDHEEDDTITYRKMDTFYFRPELSGPGLTGEETIIMPHVFMMSMAITVYRDKPSMMNMLGKAINGIFDNPSDVFMRVNAMDILFRGVIINCDRTEFAPKAACTAIKKEGAKSLIIEPNNQLRFSLFGLKNHTVDSRVVTVKRGIKNVMDVGQVVAMDGAPQLEIWNDHCNEYQGTDGTIFPPFLTQKDRLQSYSADLCRSFKPWFQKTTYYRGIKTNHYIANMGDFANDPELNCFCETPEKCPPKGLMDLTKCVKAPMYASMPHFLDADPQMLENVKGLNPDMNEHGIQIDFEPISGTPMMAKQRVQFNMELLRVEKIEIMKELPGYIVPLLWIEGGLALNKTFVKMLKNQLFIPKRIVSVIRWWLLSFGMLAALGGVIFHFKDDIMRIAIKGDSSVTKVNPEDGEQKDVSVIGQSHEPPKINM

>MsexSNMP2 (AAG49365.1) sensory neuron membrane protein 2 [*Manduca sexta*]

MLAKHSKLFFTGSVVFLIVAIVLASWGFPKIISTRIQKSIQLENSSMMYDKWVKLPIPLIFKVYFFNVTNAEGINEGERPILQEIGPYVYKQYRERTVLGYGPNDTIKYMLKKNFVFDPEASNGLTEDDDVTVINFPYMAALLTIQQMMPSAVAMVNRALEQFFSNLTDPFMRVKVKDLLFDGVFLNCDGDSPALSLVCAKLKADSPPTMRPAEDGVNGYYFSMFSHLNRTETGPYEMVRGTEDVFALGNIVSYKEKKSVSAWGDEYCNRINGSDASIFPPIDENNVPERLYTFEPEICRSLYASLAGKATLFNISTYYYEISSSALASKSANPDNKCYCKKDWSASHDGCLLMGVFNLMPCQGAPAIASLPHFYLASEELLEYFEDGVKPDKEKHNTYVYIDPVTGVVLKGVKRLQFNIELRNMPRVPQLQAVPTGLFPMLWIEEGAVMTPDLQQELRDAHALLSYAQLARWIILAAAIILAIIATITVARSTSLISWPRNSNSVNFIIGPMVNDKMR

>MdemSNMP1 (XP_008543234.2) PREDICTED: sensory neuron membrane protein 1 [*Microplitis demolitor*]

MKEMWVKVPFPVDFRIYLFNITNANEIKTGAKPIVQQVGPFFYDEWKEKVDLVDREEDDTVEYKNKATWVFNQAKSAPGLTEDVVLVFPHVMILSMILATVREKPAMVGLAAKAVDSIFHKPDSVFVTATAREILWTGLPVDCSVKDFAGSAVCGILREDDSGFLKDGENYKFALFGAKNGTVIPDTIRVHRGKRNYLEVGIVTEFKGEPKLNVWPEEGDCNTFNGTDSTIFHPFLYQDEDVVSFAPDLCRSLGARYQRPSKVKGIKTNRYTATLGDMSTDPALKCFCPTPDTCLGKGLYDIFPCVKAPLVGSLPHFYDTDPQYLTQVDGLHPNEEDHQIFIDFEPMLGAPLSARKRLQFNIFIMPVEKFKLMKTFPNALLPLFWVEEGLLLDDEYLKPIKMVFTMLKIVGIMKWLMMTAGVGLGGGAGFLFWKSTQSPQKLDITKVSPKTIQNAGGDEKKWPTTVSTIQGNNGPPSVEA

>MdemSNMP2 (XP_008549356.1) PREDICTED: sensory neuron membrane protein 2-like [*Microplitis demolitor*]

MNYCRTGFRNFLSYVPGLLLISLGIYLATEKPHTNYVIDQIRKEAELTEGKYGYRMWKDLDIYFKVYLLHVTNPDNVMEGENPIFEERGPYVYNLNMKKQVTHVDEKLDELAFTIFRTYQFNKNASGSFSEDDQVVLLNSAYLGTLNTIVSKFPAFIGRFGNSIQNLFPKTYDVFLRSKVKDILFDGLPLICDPVKYKDLALLCNFLKGQKPPIIKNTDKPGVYSYSLFDKNNYTDSDPFTVNRGVENKDALGNTTSFKKLRVTKYWTEKECNLVSGTDSITWAPMTEKLPFVSVYEPNVCRRMTPNFKREVIINGLMGYRYELDETTWLKKNMGCYCLPNAKKVPECLQTGLLDITKCQVTLLFIYITRDAPVIFSEPHFLHADPNLLEYARGLKPDPIEHTTFITIEPLSGAPLSGSKKIQLNLNVKRIPGITLLANISEGLFPILWAEEVFYLFIFINKQLSQ

>DallSNMP1 XP_015114625.1 PREDICTED: sensory neuron membrane protein 1-like isoform X1 [*Diachasma alloeum*]

MQLWLKLGIAGGCLFMFGTLFGFAIFPPFLRSQLKKQVTLKPGGDIRDMWEVVPFALDFRVFLFNITNPDEIKAGAKPIVNEVGPFFYDEYKEKVDLVDNNEEDTCEYSNKATWMFAPEKSGVGLSEDTVLTFPHVMILTLILTVVREKPGMMGLAAKAVDSIFKKPDSVFVKATVREILWTGLPVDCTVQDFQGKAVCSLLAENEAAFIVEGPGKYRFALLGAKNGTVVPDRFKVRRGIKNYRQTGEMVSYKGEERQKVWADDGPCNALPGTDSTIFHPLLFQDEDIVSFSPDMCLSLPAYYVKPSKVKGLKTNHYNADFGDMNTDENLKCLCTAPDKCLKKGLIDLFTCLGAPMVASLPHFYLVDPFYLTQVDGLHPNQEEHQIFINFEPMTATPVEARKRLQFNMFIFPIEKFKLMKTFPEALLPLFWVEEGLLLGDEFVKQLKVVFTMIKVVGVMKYLMMFGGIGLAGTSGFLKYRDMQASQKLSITKISPKATANATAPDSGEKRWPLNINTVQAQSVPALDG

>FariSNMP1 XP_011306891.1 PREDICTED: sensory neuron membrane protein 1-like [*Fopius arisanus*]

MRLWLKLGIAGGSLFMFGTLFGFAIFPPFLKSQLKKQVTLKPGGDVRKMWEVVPFGIDFRVYLFNITNPDEIKTGAKPIVKEVGPFFYEEYKEKVDIVDNEEEDTAEYSNKVTWKFNLEKTGAGLSEETVITFPHLMILSMIMTVLREKPGMIGFAAKAVDSIFHKPDSIFFKATVKEFLWTGLPVDCTVQDLQGKAICSLLAENEGAFIKEGPQRYRFALLGAKNGTVVPDRIKVRRGLKNYLEVGEVIEVKGEKKQKVWAENGPCNDYQGTDTTIFHALLYEDEDVVSFATDLCISLPAYYRAPSNVKGIPTNQYTADFGDMNKDEHLKCLCPAPDKCMKKGYIDLFPCLGAPMIASLPHFYLADPIYLSQVDGLRPKKEDHEIFMNFVALAGAPIEAAKRLQFSMFIQPVEKFKLMKTFPNALLPLFWVEEGVLLDDEYLAPLKMLFTMITIVNVMKWLMMAGGLALAGTSGFLKYKEMQASQKLTITKVSPKVTANGNAPGSTEKKWPLNVSTLQSQNVPGIDN

>MpulSNMP1 AQN78521.1 sensory neuron membrane protein 1 [*Meteorus pulchricornis*]

MKLFMKLGIAGGAMFAFGILIGFIIFPPFLKSQVKKQSSLKAGSELREMWTTLPFPLDFKVYLFNVTNPEEIAAGAKPIVKEVGPFFYDEYKNKVNLVDDEEDDTVEFSFQKTWYFNQAKSGPGLTEYTEIIFPHMLILGAVMTTLRTQPTMVGAVGKALDSVLHKPESVFMKTTPRELLWEGMLIDCTVKDGAGKALCKELRKDDSGLLKEGENYRIALFGHQNGTASRDRIKVKRGLKQILDVGVVQTFNNKTKLDTWVEKGNCNTLNGTDSTIFHPFLYDNEDIVSFSSDICRSVSARFQQKSSVGGIKTNRYTASLGDMSKDPEIKCLCPTEDTCLKKGLMDLFNCVKAPIVASLPHLYQVEKEYLSQVDGLHPNEHEHELFIEFEPFTGSPLSARNRLQFNMFIQNVTKFKLMKTFPSALMPLFWIEEGILLGDDILSQIKMVFTLMSVVSGMKWTMITLGLGLGGAAGGLFYKAQQNSQKLDITKIVTKSSQNGGEKQWPVNISTVQGATAQPHLDS

>PcanSNMP1 XP_014608006.1 PREDICTED: sensory neuron membrane protein 1 [*Polistes canadensis*]

MELIKKLGIGGGVLFFLGITFGWMGFPALLKSQIKKAIALKKNSEMREMWSQFPLPLDFKIYLFNVTNPKEIAQGEKPIVKEVGPFFYDEYKEKVDLVDREEDDSLEYSLKATWFFNPSRSNGLTGEEELVLPHLLILAMVVTTMREKPAAIGILNKAVDSIFKKPDSVFVRVKAREILFDGLPVDCSVKDFAGSAVCSILKSEGKDLMNDGDDHYRFAIFGAKNGTVMPERLRVLRGIKNYKDVGRVLEFDKKPALTIWTEDHCNEFNGTDSTIFPPLMTKEDDIVSFSPDICRSLGARFSHETKVKGVNTFHYTANLGDMSTNPREKCFCPAPDNCLSKNLFDLTKCVGAPLIASLPHFYLADEKYIRDVDGLHPNQEEHDISMDFEPMTATPISAHKRLQFNIQLRPIEKFKLMKNFPEVLFPLFWVEEGILLDDQFVKKVKVVFTAISVVGFMKWLMVLGGIGLGGTAAGMHFKRKNSENKLDITKVTPQSDSRKDSSNEKKWQTMNISTIQAATVPSSLDRY

>PdomSNMP1 XP_015183731.1 PREDICTED: sensory neuron membrane protein 1 [*Polistes dominula*]

MELVKKLGIGGGVLFFLGITFGWMGFPALLKSQIKKAIALKKNSEMREMWSQFPLPLDFKIYLFNVTNPKEIAQGEKPIVKEVGPFFYDEYKEKVDLVDREEDDSLEYSLKATWFFNPSRSNGLTGEEELVLPHLLILAMVVTTMREKPAAIGILNKAVDSIFKKPDSVFVKVKAREILFDGLPVDCSVKDFAGSAVCSILKSEGKDLMNDGNDHYRFAIFGAKNGTVMPERLRVLRGIKNYKDVGRVLEFDNKPALTIWTEDHCNEFNGTDSTIFPPLMTKEDDIVSFSPDICRSLGARFSHETKVKGVNTFHYTADLGDMSTNPREKCFCPAPDNCLSKNLFDLTKCVGAPLIASLPHFYLADEKYIREVDGLHPNQELHDISMDFEPMTATPISAHKRLQFNIQLKPVEKFKLMKNFPEVLFPLFWVEEGILLDDQFVKKVKVVFTAISVVGFLKWMMVLGGIGLGGTAAGMHFKRKNSENKLDITKVTPQSDSRKDSSNNEKKWQTMNISTIQAATVPASLDRY

>OabiSNMP1 XP_012278791.2 sensory neuron membrane protein 1 isoform X1 [*Orussus abietinus*]

MSHVSAFSRILQGALSWFLRTSRTFLRQCERVLYFFQVNSNKNNAFQKKRSFLEKLVSDFSGALRYVKKSGSKLLGESLVSEMKLPNKLAIAGGILFSIGVMFGWVIFPMVLKSKINGAIALKKGSEIRELWSKFPLPIDFKIYIFNVTNPEGIQKGEKPIVKEIGPYFYDEYKEKVDLEDRDSDDTVEYKQRVTWIFNPSKSNGLTGNEELIFPHLLMLGIVMGTLQQQPAMMGIVGKALDSIFKKPDSIFIKVKVKDVLFDGLPIDCTVKDFSGSALCSKLKEQGKDLVVEGENRYRFSMFGHKNGTIGEDRLRVVRGTKNAKDVGKVVEFNGQPSLTTWPEEHCNAYNGTDSTIFHPFLYKDEDVVSFAPDLCRSLSARFDSPSAYKGIRTNRYTADLGDMSTHPEEKCFCPTEDTCLKKGLYDLTKCVGAPIVASLPHLYLADESYLETVEGLKPTQEDHQIFLDFEPMTGTPVSARKRLQFNMFIQPVQKFKLMKTFPEAILPLFWVEEGLALNDDFVGKLKAAFKMIKIVGYIKWMLVITGTGLGIATGILHFKDRHSKSSLDISKVSPTAGNKMANGQEKPWPLNVNTLQSAPVPATLDGS

>ArosSNMP1 XP_012257194.1 sensory neuron membrane protein 1 isoform X1 [*Athalia rosae*]

MELPKKLGIGGGVLCFLGIIIGWLAFPKLLKSQINGAIALKSGVEMRDMWSKLPFPLEFKIYLFNVTNPADIANGEKPIVEEIGPYFYDEWKEKVNLVDRDEDDTVEYNQRITWHFNASRSNGLTGDEELVFPHLAILGMMMITVSEKPTMVGVAAKAVDSIYKKPSSVFVRATAKEIMFDGLPVDCSVKDFAGSAVCALLKAQGKDLVPLGDDKYKFSIFGHKNGTISAEKMRVYRGYKNPKDVGRVLEWKNESALTIWAGDECNAFNGTDSTIFHPFLYQDEDVVSFSSDICRSLSARFQHPTEVQGIRTNRYSADFGDQSSVPEEKCFCPTPETCLKKGAMDLYKCVGTPVVATLPHFYLADPSYLETVSGLHPVKEDHEISIDFEPMTGTPLIARKRLQFNMFIMPIEKFKLMKNFPNALLPLFWVEEGVELGDDIIKKLKGAFKLITIVTCVKWTMVFLGLGLGSAAGFLEYKRRQLTDKLEVTPIPAGGTIRTTISAGKNGDEKKWPPNISTLQAAAVPATLDGSF

>CcinSNMP1 XP_015587038.1 PREDICTED: sensory neuron membrane protein 1-like [*Cephus cinctus*]

MQLSTKLGIGGGALFVMSIVVGWIAFPIMLKSQVKGAIALKEGSDMRALWAKFPLPLDFKVYLFNITNPAGIESGEKPIVREIGPYFYDEYKEKLNLEDRESDDTVEYSQRATWFFNPSKSNGLTGDEELVYPHVLILGMAMTAARDKPGMMGLLGKAVDSIFKKPDSIFIKVKARDILFDGLPIDCTVKDFAGTAVCKLLKEQGNDLQVDGDNRYRFSLFGHKNGTVARERMRVFRGIKSAMDVGRVVEWNNEPALTVWPEDRCNEYNGTDSTIFHPFFKKEDDVVSFAPDLCRSIGARYERPSHYKGLSTNRYTANLGDMSTDPHLQCFCPTPETCLKKGLFDLTKCVNAPIIASLPHFYLTDESYLETVQGLQPVQEDHEIFIDFEPLTGSPVSARKRLQFNMFVYPVEKFRLMKTFASALLPLFWVEEGIALNDDFVKQLKAAFKMISLVGYIKWSMMSLGLGLAGAAGGLYFKRRQTDNKLTINTITPQMASTKNSGEEKKWPLNINTLQAATVPASLDRN

>LhumSNMP1 XP_012225459.1 PREDICTED: sensory neuron membrane protein 1-like isoform X1 [*Linepithema humile*]

MVSKRRAYDVTVRTLRRIHDTLRRTKLLYAFPFGSAFDKVLRVLEEFNKSTKMVNIKKLGIGGGCMFVFGILFGWWGFPAILRSQIKKAIALKPGSEIREMWSNFPLPLDFKVYLFNVTNPDEIKEGKKPKLQEVGPFFYDEYKVKLDLVDREEEDSVEYSLKSTWFFNPKKSNGLTGEEEMVYPHLMILGMVGATLIEKPAAVGIVGKAVDSIFHKPDSIFVRAKAKDILFDGLPVDCTVTDFAGSAVCNLLKTEAKDLVPDGENRYKFSIFGGKNGTIAPQRMKVLRGVKNYKDVGRVLEYDGKPALDIWPEDHCNAFNGTDSTIFPPLFGPDDDIVSFGYEICRSLSAHYKHHSKIKGVNTLHYTADLGDMSTNPMEKCFCPTPDTCLPKNLYDMTKCLGVPIIGSLPHFYDSDGKYLQMVDGLHPNQEEHEIDMDFEPMTATPLLAHKRLQFNMFIQPVPKFKLMKNFPEALLPLFWVEEGILLDDEFVNKVKVVFKAMAVVGFLKWLMVLGGLGMSVAAGVLHYKSRDSGKLDITKVTPKGSSKEDGKADKSWPPSMNISTIQAASVPSSLDRN

>HlabSNMP1 XP_017795434.1 PREDICTED: sensory neuron membrane protein 1-like [*Habropoda laboriosa*]

MKPKKLGIIGGSMLAFGVLFCAVIFPPFLRSQIKKQVALKDGSEMRDLWSNFPLPLDFMIYLFNVTNPTEIMAGGKPILKEIGPFFYDEYKQKVDLVDREEDDSLEYGLKATWYFNPSRSNGLTGEEEIVFPHVLILSMIKVTLMEQPAAIGILNKGVDSIFKKPNSVFVRAKVREILFDGLPVDCTVKDFAGSAICSVLKTKPDALIPDGEGRYLFSLFGPKNGTVLPERIRVLRGIKNYKDLGRVTEFDGKPALSLWTADHCNEFNGTDSTIFPPLLTEEDDIVSFAPDICRSLGAHFTEKTKVKGVNTYHYKADLGDMSTNPMEKCFCPTPDTCLTKNLMDLFKCVGAPLIASLPHLLGSDEKYRQLVDGLHPNEEDHGISMDFEPLTATPISAHKRLQFNMFLHPIEKFKLMKKFPECLFPLFWVEEGILLDDQFVKKVKTVFMAISVVGFLKWLTILGGICVSGAAAAMIFKNRGKGSLDITKVTPQSQNGKDGEQKKWPNQMNISTIQSAAVPPNLDAN

>TcorSNMP1 XP_018359576.1 PREDICTED: sensory neuron membrane protein 1-like isoform X1 [*Trachymyrmex cornetzi*]

MVTKRRAYDVAVRELRRMYDVLKRSKLLYAFPFGKTFDNVLRVLEEYNKGVKMVSIKKLGIGGGVSFIFGILLWCGFPALIKSQIKKAVQLKPGSEIRDMWSVVPFPLDFKVYMFNITNPDEIKEGKKPRVQEVGPFFYDEYKVKFDLVDREDEDSVEYSMKTTWFFNPKKSNGLTGEEIMVFPHLMILGMVGATLLDKPAAIGVVGKAVDSIFHKPDSIFVTAKAKDILFDGLPVDCDVKDFAGSAVCNLLKTEAKDLIPDGENRYKFSLFGGKNGTGVPERMKVLRGVRNYKEVGQVLEFNGKPALDIWPEDHCNAFNGTDSTIFAPLFGPDDDVVSFGYEICRSLSAQFERHTKVKGVKTLRYTANLGDMSTNPMEKCFCPTPETCMTRNFYDMTKCLGVPIIGSLPHFYDTDGKYLEMVDGLHPNQDEHEIDMDFEPMTATPIRAHKRLQFNMFIQPVAKFKLMKNFPNALLPLFWVEEGVLLGDEFVNKVKMVFKVMAVVNFLKWLLILGGIGLGGAAGFFYYKNRDSGKLDITKVTPKTSGDNKPEKSWPPGMNISTIQAASVPSSLDRN

>DquaSNMP1 XP_014467429.1 PREDICTED: sensory neuron membrane protein 1-like isoform X1 [*Dinoponera quadriceps*]

MSQRKLRVFDRIVVGASDCRDATPGQPGKIEVHDGAGQARSTPTPSRCKTSVSLSRGQSATSRARSTATRVARKLARRAHSLLPNVTERLTFPQTVKMVSIKKLAIGGAAMFLFGILFGFVGFPKLLRSQIKKAIALKPGSEIREMWTAFPLPLDFKVYMFNVTNADEIMQGGKPKLQEVGPFFYDEYKVKLDLIDREDDDSVEYSMKSIWYFNKKKSNGLTGDEDMVFPHLMILGMVMATLRDKPAAVGVVGKAVNSIFHNPDSIFVKAKAKDILFDGLPVDCTVTDFAGAAVCNVLKTEGKELIPDGEGRYKFSLFGGKNGTVVPERMRVMRGIKNFKDVGRVLEFDGKPALDIWPEDHCNQFNGTDSTIFPPLFGPDDDIVSFGFEICRSLSAHFSHHTKVKGVNTLRYTANLGDGSKNEDEKCFCPTPETCLTRNLYDMTKCLGVPIIGSLPHFYDSEEKYLTMVDGLNPKQENHEIDMDFEPMTATPIRAHKRLQFNMFVHPVPKFKLMKTFPEALLPLFWVEEGILLDDQFVNKVKIVFKAIIAVNFIKWLSVLGGIGMGCAAGALYYKNRDTGKLEITKVTPKLDSRADSKMEKNWPPGMNISTIQAASVPPSLDRN

>AdorSNMP1 XP_006609952.1 PREDICTED: sensory neuron membrane protein 1-like isoform X1 [*Apis dorsata*]

MHFKKLIYDVTTRFFHRGVHVILHSSFVSLQSFVTTGRKLHETLRVPVGCKRVTMKPKKLGIIGGSLLTFGILICAIAFPPFLKSQVKKQIALKNGSEMRELWSNFPIPLDFKIYLFNVTNPMEITAGEKPILEEVGPFFYDEYKQKVDLIDREGDDSLEYNLKATWFFNPSRSEGLTGEEELVVPHVLILSMIKLTLEQQPAAIGILNKAVDNIFKKPSSVFIRAKAREILFDGLPVDCTGKDFATSAICSVLKEKDDALVADGRGRYLFSLFGPKNGTVLPERIRVLRGIKNYKDVGKVTEVNGKTKLDIWGEGDCNEFNGTDSTIFAPLLTEQDEIVSFAPDICRSMGARFDSYTQVKGINTYHYKADLGDMSSHPEEKCFCPSPDSCLTKNLMDLTKCVGAPLIASLPHLLGAEEKYLKMVDGLHPNEEEHGIAMDFEPMTATPLSAHKRLQFNLYLHKIEKFKLMKNFPECLFPVFWVEEGILLSDEFVKKLKTVFKTISIVGFMKWFTIVSGTCVSGAAAALFFKNKDKNKLDITKVTPKKSEEKKWPNQMTISTIQSAAVPPNLDAD

>AcerSNMP1 XP_016917631.1 PREDICTED: sensory neuron membrane protein 1 isoform X1 [*Apis cerana*]

MRFKKLVHDVTTRFFYRGVHVILHSSFVNLQSFVTTGRKLHETLRVPIRCKCITMKPKKLGIIGGSLLAFGILICAIAFPPFLKSQVKKQIALKDGSEMRELWSNFPVPLDFKIYLFNVTNPMEITAGEKPILEEVGPFFYDEYKQKVDLVDREEDDSLEYNLKATWFFNPSRSEGLTGEEELVVPHVLILSMIKLTLEQQPAAMGILNKAVDNIFKKPSSVFVRAKAREILFDGLPVDCTGKDFATSAICSVLKEKDDALIADGPGRYLFSLFGPKNGTVLPERIRVLRGIKNYKDVGKVTEVNGKTKLDIWGEGNCNEFNGTDSTIFAPLLTEQDEIVSFAPDICRSMGARFDSYTQVKGINTYHYKADLGDMSSNPEEKCFCPTPDSCLTKNLIDLTKCVGAPLIASLPHLLGAEEKYLKMVDGLHPNEEEHGIAMDFEPMTATPLSAHKRLQFNLYLHKVEKFKLMKNFPECLFPIFWVEEGILLGDEFVKKLKTVFKTISIVGFMKWFTIVSGTCVSGAAAALFFKNKDKNKLDITKVTPQKSGEKKWPNQMTISTIQSAAVPPNLDAD

>DallSNMP2 XP_015116225.1 PREDICTED: sensory neuron membrane protein 2-like [*Diachasma alloeum*]

MKLTSHGKQQLSIDPHKYSMKNVTAITKDIELREGTDNYIAFLKIKVFFRVWLFEVTNPEAVMAGENPLLKEVGPFVYDLHIKNHINRIDEASDEINFTVTKTYYFNQQESGDLSEDTVVTVLNFAYLGTIKKIISLAATFLKKIGPIIHQIFPGAVNPFLTGKAGDIIFSGLPLDCVNVDKALNMICNVLKGNPPALLKKTDTPGLFKYSLFYRLQVNGTHQGPFTVNRGVKNIYSLGNTTSFKNMKVTNFWATDTCNAVSGTNAITWPPMSKKLPKVQSYEAQLCRSLSPSFTREVVLNTLAGYRYELTADTWNQEEMECYCPRNKKKVMECLPLGLMDIEKCQEVPIIFSEPHFLHASSELLDYAQGLHPIHEKHATFVVIEPFTGVPLSGSKKIQLNMKMSSIPTVPWLTNVTDGYFPILWAEEVSPSLLSDPKQNPSGNSPISHVSVQTSSSRIPQSYLRLESLWFFLALN

>HlabSNMP2 XP_017796759.1 PREDICTED: sensory neuron membrane protein 2-like isoform X1 [*Habropoda laboriosa*]

MFCYHICGAVCIVIGIFIVSRQFMWTEVRKNILTKLPLIEGTETYKVWLSPTLLTFGCYLFNVTNPDGVMRGEKPHLAECGPFMYDEIYGRYVLDVDKETDEIKYVTKSLYSFNKESSMTVSRQDKVTILNPAYVGTIALLATLPPDILAKYGNHIPKLFPNRSSIFLTARPTDILFDGLKVTCNLKKYPELDKVCKTLKINVPPVLRTTDREDVYLLSLFQRMNDTYRGPFSMNRGLKNITRLGDTTSYKGKRVQTVWNSDKCNTVRGSDTITWPPLIEPLPFVSTFIPDLCRSIEADYEGDAWVHGLLGSRFVMKERMWDLNETECYCLPLNHVPQCLPQGLLDVSECQKVPIIFSEPHFLHADPELLTYARGLRPNKRIHETYIIIEPNTGTPLSGSKKMQLNLKLTKQRVDLLSNVSEGYFPMLWCENGNTPTLAVISFTYQILRMANIIWFMEKVPLIVGIYILLMSTLFCDCTKRKVKPTISPAESILISSYSRSDSDAHIAPRRHAHAFQ

>CcinSNMP2 XP_015590128.1 PREDICTED: sensory neuron membrane protein 2-like [*Cephus cinctus*]

MGKRVNWYNLLWYLVGLLLLGTGIFIIIKKPHTDIVLNFVHKKNMLVPGNRIYDIWQNLEFDFQVYLFHVTNPAKVMKGEPPQLVEYGPYVYDEHLYKEVIKVDEERDEIEYMLKRKLVFNKEKSNGNSDEEPITILNSGYLGIMLMINSLLPSMVSTMGADVQKLFPKLRDIFSRGKVKDILLDGIPFVCDVQKYPELLSICNIVQGKPPPAIKATDTPGHYLFSLFGKINQFPVGPFTVNRGVKDWRRRGILTSFKNSEYLTYWALPDCSKISGTDSVVSPPMTTPEPEISSYIVDLCRSVNVKYNGTVDILGLNGYSYTKVNSSWEGNETDCYCPKIKQETKCPLYGLMDVTKCQEAPVIVSEPHFLHGAQELLLYAKGLNPDEERHKTFVIMEPLTGIPLRGFKKMQLNMYLQKLPVKLLSNVSEGYFPFAWVAEGKMEHAELLIDVIQLHRLLRLFDFICWLPSMCAIFIIFATCCLHNVRKSTKNVVPVLRYSLTVSPIAHARN

>BterSNMP2 XP_020718450.1 sensory neuron membrane protein 2-like [*Bombus terrestris*]

MWCYYGCAIIWVIFGLYLSQTEVFLNKFISKITETISLREDSELYKVWMSPIHLTFTCHFFNVTNPDEVMSGSNPYFNEVGPFTYDEILEKQIIDVDDTMDEITYTTKSTYSFNKDLSVKLSKHDKITILNPAYIGTISMLSGLPPEFMEKYGNNIPKLFPNRSTIFLKANPKDLLFNGIKISCNLRKFPELDLICKTLGSNPPLVLRETDKQDVYLLSIFQRMNATFRGPFSVNRGVNDITKLGDITSYMGKRIQTMWNSEDCNTVRGTDSIIWAPLIKPLPFVSTFIPDLCRTIEADYKDEVSVRGLIGSRFVMKERTWFLNTSQCYCLLENKIPKCLPQGLIDVSECQKLPVVLSEPHFLHGDPQLLKYARGLNPDERLHETYIIIEPYTGTPLSGQKRTQINLYLEKQSVELLSNVSEGYFPLMWCENMLTYEYNLSLHHAPDHFKM

>AcerSNMP2 XP_016914160.1 PREDICTED: sensory neuron membrane protein 2-like [*Apis cerana*]

MWSYQVCAIICLIFGIYVFITNMFSEGLFSIKNNIFKNLPLVKGKDMYNEWILPVSLIFKCYLFNVTNPDEVMQGDNPNLVEYGPFTYKEVFEKQIVDVNEELDEIIYDVKSTFRFDKYASLNISKRDTVTILNPAYIGTISMLNTLPPTYMEKFGNNIPKLFPNQKSIFLKANPKEILFDGVKLTCNERKFPELNMICKTLKTLRSPVLKEGEKDGVYYLSVFQRINGTIRGRFSVNRGVNNITELGNISSYNGRRVQTIWRTEKCNTVRGSDSITWAPLVNPLPSVLTFVSDLCRSIETDYDKKVSIYGLIGYRYVMKERTWFLNTSQCYCLEKNKVPNCLPQGLIDVTDCLKIPIIMSEPHFLHGDPQLLMYARGLNPNEDEHETFIVIEPYTGTPLSGQKKIQLNLKLERQPVDLLSNISEGYFPLLWCANVRIFSKIIILQH

>BimpSNMP2 XP_024226005.1 sensory neuron membrane protein 2-like [*Bombus impatiens*]

MSPNNRQTDNFKEKVRSNFYKMWCYYGCAIIWIIFGLYVSQTELFSNKLISKITEALSLREDSKIYKAWKSPMQLTFTCHFFNVTNPDEVMSGSNPYFNEVGPFTYDEILEKQIIDVDDAMDEITYTTKSMYSFNKDLSVKLSEHDKITILNPAYIGTMSMLSSLPAGYIEKYGNNIPKLFPNRSSIFLKANPNDLLFNGIKVSCDLRKFPELDVICKTLGSNLPQVLRKTDKQDVYLLSIFQRINATFRGPFSVNRGVNDIMRLGDITSYMGKRKQKIWNSEDCNIIRGTDSIIWAPLIKPLPFVSTFIPDLCRTIEADYKDEISVRGLIGSRFVMKERTWFLNTTQCYCLLENKIPKCLPQGLIDVWECQKLPVILSEPHFLHGDPQLLKYAGGLNPDDRLHETYIIIEPYTGTPLSGQKRMQLNLYLGKQSVELLSNVSEGYFPLIWCGNVRSFKTVPIILSCLLNIYDFTYEMQLIKIIRFNSQDISFIMNLL

>DnovSNMP2 XP_015438309.1 PREDICTED: sensory neuron membrane protein 2-like [*Dufourea novaeangliae*]

MLAKLSEDPDKWFKILDKVEFALNNSVCRSTAETPSRLLFGMNQLGLVNDKLRLELDANISVERDLSAFRDAAFRKIEKCQLENERLYDWKRKPAIEYQVGDNVVISNHVVAPGVNRKLLPKFKGPYVVTKEVVLIKGTAAYEAWISPVHLTYACYIFNVTNPDEVMQGENPNLIEYGPIVYDEVMKRNILDIDEEADEIKYTTKAMFTFNRHKSVNVSSSDKVTMLNPAYVGTILMLSSLPPNFMQKYGNSIPKLFPNRSSIFLKARPQDILFNGVKVSCNEKKFPELNLICKTLKSKRPPVLREGAKEGVYLLSMFQKINNTIHGPYTVNRGVKNITLLGDTTSYMGERVQQIWGSESCNSVRGTDTITWPPLVTPLPFVSTFIGDLCRSLQADYDSDISIHGMTGSKFVMKERVWYLNESQCYCPVVNKKVECLPLGLVDLSKCQEAPVIFSEPHFLHGDVELLTYARGLKPVENLHSTYIVIEPHTGVPLSGAKKSQLNMKLTKQPVDLLSNVSEGYFPLVWCEEGSTPTLKVVGLSYQTMRSVRLAMFLQRLPLIVGIYMVSVSLFLCKNTRSKVEPTRSMIRSTLMSSDQQPYNYSHRPPRWHTNAFERFQRM

>EmexSNMP2 XP_017753098.1 PREDICTED: sensory neuron membrane protein 2-like [*Eufriesea mexicana*]

MYCYRVCAVVWIIFGIYTIITRTFRHGIVKEILTFLPLNGDGETYDAWVSPMHLTFACYLFNVTNPDEVMRGSYPNLAEVGPITYDEIYEKQVMSVDEESDEITYITRSTYTFNKYLSLNITKRNKINILNPAYIGTISMLSSLPPDIMKRYGNDIPKLFPNRSSIFLKASPMEILFDGVKVSCNLKKFPELDMVCKTLISNPPPVLRETDREGIYLLSIFQRMNYTFRGPFSVNRGLKDITRLGDITSYKGKRVQTLWSSDSCNTVRGTDTITWAPLVEPLPFVSTFVPELCRTVEADYEDEVSIHGLTGSRFVMKERTWLKNETECYCVEVNHVPQCLPQGLIDVTECQGVPVILSEPHFLHGDPQLLTYAQGLSPDESVHGTYIVIEPYTGTPLSGEKKTQLNLELKKQPVKLLSNVSEGFFPLMWCGNVSSFLKIIYQFCFGLFQTVEADYEDEVSIHGLTGSRFVMKERTWLKNETECYCVEVNHVPQCLPQGLIDVTECQGVPVILSEPHFLHGDPQLLTYAQGLSPDESVHGTYIVIEPYTGTPLSGEKKTQLNLELKKQPVKLLSNVSEGFFPLMWCGNGNTPSLSIIGLTYQVHRLIQVISYLDIVPLMIGIHMALMTTLYCNCTRRKVQPTVSIADSLLISSNSQSNNSTHRPRRQAVQAFEQF

>MrotSNMP2 XP_012141978.1 PREDICTED: sensory neuron membrane protein 2-like [*Megachile rotundata*]

MSNLYLMIFQIFIGISLICLGSYFVSENVLETLVVKFVQKFSLPLIKGSPHYEIWSKPTSINFSCYLFNVTNPDEVMRGENPHLVEYGPFTYTEVQEKFISYIDKEMDEIKYTTKSTYTFDRYQSLNFSKQDKIIILNPAYIGTIETLAALPDDFMKKYGNSIPKLFPNRSSIFLKARPTDVLFDGVKITCNPKKFPELQLICETLKLKQPPVLREGEKENVYYLSFFQRLNNTSRGPFTVSRGVKDITKLGDITSYLGYRVQEVFATDLCNTVRGSDTITWAPLMKPLPKVSTFIQDICRTVEIDYENEVVLNGMIGSQFVMHERVWYLNESECYCPLVDKQPVCPRRGLIDAYQCQKVPVFVSEPHFLHGDPELLNYARGLTPNEVLHKTYVVIEPYTGIPLAGEKKTQLNLKLARRPVNLLANISEGYFPLLWFENGNKLTPFDQVLYPYQLLRYLHFMRYLQYIPLIIGIYLISISLLFYGDTSRRVHPNATNVQSILISNSRLNRPPQRQVVW

>ArosSNMP2 XP_020706322.1 sensory neuron membrane protein 2-like isoform X1 [*Athalia rosae*]

MNTAMQLLLRCDTISNRVVRDIITPGNSLDIILKKADSNYKTLQFIQDCSVMRGLGSPSYSMGFGIAFVIVGTIAMVIRVSNRITEHVLDSASCLKEGSLGFKLWKTVDLQFKAYLFNVANPTEVQEKGDAAILVEYGPYVYDEHIEKIVIGVDESTDTIEYMVLKTLFFNGTKSGNLSETDEVTILNPAYLGIIHTLANMFPAFVGRFGNSIPLLFPDSNSIFVKGQVKDILFDGLPLICDSATHRELNLICSFLKTKRPPILRTTPDAGVYAYSFFNKINSTAEGPYTAFRGTKDRFRAGDLSKFKGSAYSDDGSMKNCSIIKGSDSLYWPPIKSRESTLFSFISDLCRSLPSEYTKDDFHSGLMGHRYSPSENMWNGSDSSCYCTMAPNGSANCPTRGLLDLTKCQGAPILMSWPHFLYGAESILSSIRGIRPDPKKHAAYVIIEPLTGTPLVGTKKMQLNMILQQQQVRLLANVSRAVFPLVWVDEGLTVPTKLLAPIVVVHRVRRVLEFITWILLLFGILLIVHSCWMNAGRRYIGGSSGGIILPPENTLN

>CcalSNMP2 XP_017881163.1 PREDICTED: sensory neuron membrane protein 2-like [*Ceratina calcarata*]

MGLKSISLVEGTHVYKIWASPITLSFACYFFNVTNPDEVMQGEHPRLVEYGPLTYDEIYEKQVIDVDEEMDEIKYTTKSTYTFNKAKSIDISNREKVTILNPAYIGTISMLSSLPPNYMEKYGDHIPKLFPDRNSIFLKARPTDILFGGVKISCNLKKFPELDLVCKTMNASPPIVLRKTEKEDTYLLSLFQRSNNTLRGPFTINRGFKVITSLGNITSYQGEKIQSIWNSDVCNTVRGTDSVNWAPLIEPLPFVVSFVPEMCRAMEADYSEDVSILRMTGSRFVIKERVWFLNASECYCLLKDKNPRCLPQGLIDVTECQKVPAILSEPHFLHGDPELLMYAHGLQPDENLHATYITIEPLTGTPLFGTKKSQVNLELTKQPVKLLSNVSEGFFPIVWCENVRFLQISTKFLFSQY

>AdorSNMP2 XP_006614718.1 PREDICTED: sensory neuron membrane protein 2-like isoform X1 [*Apis dorsata*]

MMNLPLVKDKDTYNAWISPVSLIFKCYFFNVTNPDEVMQGANPNLVEYGPFTYREVYEKQIVDVDEEFDEIIYDIKSTFTFDKYASVNISKRDTVTILNPAYIGTISMLTTLPPNYMDKFGNNIPKLFPNRNSIFLKANPKEILFDGVKLTCNEKKFPELSTICKTLKALRSPVLKEGEKDGVYYLSIFQRVNGTIRGRFSVNRGINNISELGNIASYNGKKVQTIWKTEKCNIVRGSDTITWPPLINPMPSVLTFIPDLCRSVEVDYDKKVSIYGLTGFRFAMKERTWFLNTSQCYCLEKNKVPNCLPQGLIDVSNCLKVPIIISEPHFLHGDPQLLMYAHGLNPDKDLHETFVVIEPYTGTPLSGQKKIQLNLKLERQPVNLLSNISEGYFPLLWCSNGNTPDISVIIFTFQLLRLIKLIKFIDVVPLMIGIHMTIISMFYCNCKKRKRQSTISIADSLLISSNSQSNIAWRST

>MquaSNMP2 KOX75036.1 Sensory neuron membrane protein 2 [*Melipona quadrifasciata*]

MVRYIIKLGLYSKNNYSIDSYKGTISQILPLVEGSPIYKAWSSPIHLTFKCYIFNVTNPDEIMQGSNPNLDEVGPFTYNEVLEKQIISVDDEMDEIVHTLKSTYSFNKHLSLNLSKSDKVTILNPAYIGTISMLAGLPPDFMEKYVKVSCNLRKFPELNLVCKTLNNNQPPVLRKTDKEDTYLLSIFQRINGTFRGPFSVNRGLKNITRLGDITSYMGKRVQTTWNSESCNTVKGTDTITWPPLIEPLPFVSSFVPELCRTVEADYAGDVSVRGLTGSWFVMKERVWSLNTSQCYCLKVNNVPKCLPQGLIDVSECQKMPVILSEPHFLHGDPQLLKYARGLSPNEETHATYIIVEPYTGTPLSGQKKIQLNLYLERQPVELLSNVSEGYFPLLWCENGKIGFTYQIIRLLNVLRYLSIVPLTIGIHLTFVALLYCSCTKRKIGPTFAESLLISSNSQSNNSTQRPLHA

>NlecSNMP2 XP_015515345.1 PREDICTED: sensory neuron membrane protein 2-like [*Neodiprion lecontei*]

MYRREARCELLEKIVIETDDAADTIDYLGRRTLVFNATKSGNLSENEEVTILNAAHVGSLITVRMQVHNRRLCDLQRSEEIVAFRITIANGFPALLSKFGNGLAQLFSDPTSIFMRGRVRDILFDGLPLVCDPKAHPELVLVCGLLKRQRPPTLRTTSEAGIYAFSFFHKINGTDDGPMTVSRGVNDRYTTGHLSEFKGLPYADFWSIKNCSIIRGTDSIFWPPMSSRQPEVFTYINDLCRSVFAVYKNDTVYSGLLGYYYTTTESLWNDTESPCYCPREKKKIVCPTQGLLDLNKCQEVPVMMSEPHFLHGDQSLFSITNGLQPDEEKHATYVVVEPLTGAPLSGYRKMQLNLKLPRLKQIHVVANVTEGVFPLLWVEEGTTPGPKILAPIILVHRAIRLMTFLSWMPLLFGIFFVAYSYCTKTSAKTENPKASGRSTSLAAH

>PcanSNMP2 XP_014614558.1 PREDICTED: sensory neuron membrane protein 2-like [*Polistes canadensis*]

MTYTINIFHVENPDDVLQGELPRLMERGPYVYDIIIEKEILSVNEIRDEITYNIKKTYHYNDERSGELSENDEVVILNLAYLGAINTIAGFSLSLLKQYGIAIDKLFPEKNPLFVKAKVKDLLFDGIPLFCDITKHEKMKLICLTLKGKKPPILWNTDVENYYMYSILGTRNATWFGPMTSNRGVQIPTLLGEITSVNNKRAQKYWSSEECNAIKGKDSLIFPSYEEPPQRIYIYIYELCRNEKFKGMYDVWRYTNTIENWSSNQSDCYCLVSKNEKKCPPSGLMDANKCMKIPTFISEPHFFNADPSVLDYLPDLSPCEELHSSFILIDPLSSNPLEGYRKSQVNMKVTSYPIDLLANLTDGYFPIIWMSEV

>OabiSNMP2 XP_023288801.1 sensory neuron membrane protein 2, partial [*Orussus abietinus*]

MCIIEREAVKHDRNGMSSHSSNCIPDVQHPDILPVRMCRTTITSIATQFPIFLTKFGKDIVKLFPEGTDVFIHGKVKNILFDGLPLICDKDKYPEIGLMCTYLKMKKPPIIRSTDNEKLYLYSFFDKVNGSDQGSFSINRGSQNYSLLGNIVASKGNKYNKIWNTEECNRVWGCDAITCAPLEARVPHIQTYAKEYCRILQIDYSKDTRFFGTTTYRYEMMGNTWTHNASVCFCPKNLKKEIQCPPMGILDVSACQEIPIYASEPHFLHGDPKLLEYAIGLRPNELLHHTYLDIEPISGIPFSGFKKCQLNMHLAPQPVQGLHNVSEGYFPLVWIEEGANITIEQALTVVNVHRLMRFLNVLSWTPLLLGIFFILANLWLRDPVNSGLIERSSVSSQDPLFSRSKMIN

**Supplemental material 2**

SNMP cDNA sequences used in intron insertion sites alignment: Intron insertion sites (in cDNA sequences) are marked in yellow (the first nucleotide of an exon).

>*MmedSNMP1*

ATGCTTTTATTCAAAAAACTTGGTATTGCCGGTGGTTCGGTATTTACTTTAGGTATAATAATTGGTTACGCGTTTTTCCCGCCATTTTTAAAGAGTCAAATTAAAAAGGGAATACAATTGGTCGATGGCTCAGACATGAAGGAAATGTGGGTTAAAGTACCTTTTCCATTAGACTTTAGAATTTATTTATTTAATATTACTAATGCAAATGAGATAAAAGCTGGCGCTAAACCAATCGTCCAACAAGTGGGCCCATTTTTTTATGAGGAATGGAAAGAAAAAGTTGATTTGGTAGATCGTGAAGAGGACGATACTGTTGAATACAAAAATAAAGCAACATGGGTATTTAATCAAGCAAAGAGCGCTCCTGGTCTTACTGAAGATGTTGTATTGGTTTTCCCTCATGTTATGATTTTGTCAATGATACTCGCGACTGTCAGAGAAAAACCAGCGATGGTTGGTCTTGCGGCCAAAGCTGTCGATAGTATATTCCACAAGCCAGATTCGGTGTTTGTCACAGCAACAGCACGTGAAATACTTTGGACAGGTTTACCAGTCGATTGTTCGGTTAAAGATTTCGCTGGTAGCGCAGTGTGTGGAATACTTCGTGAAGACGATTCAGGTTTTTTGAAAGACGGCGAAAATTATAAATTTGCATTATTTGGCGCTAAAAACGGAACTGTTATACCTGATACAATTAGAGTACACCGTGGTAAAAGAAATTATTTAGAGGTAGGAATAGTTACGGAGTTTAAAGGTGAACCGAAATTAAATGTCTGGCCTGAAGAAGGAGACTGCAATACTTTCAATGGCACTGATTCAACTATTTTCCATCCATTCCTTTATGAAGACGAAGATGTCGTATCTTTTGCGCCCGATCTCTGTAGAAGTTTGTCTGCGATATATCAAAAGCCCACTAAAGTTAAAGGAATTAAGACAAATCGGTATATCGCAAGTTTGGGTGACATGAGTACGGATCCAACTCTCAAATGTTTGTGTCCAACACCGGACACTTGCTTAGGAAAAGGATTGTATGACATATTTCCTTGTGTAAAAGCACCACTTGTCTGTAGTTTACCACATTTTTATGATACTGATCCACAATACTTAACTCAGGTTGATGGTCTTCACCCTAATGAGGAGGATCATCAGATTTTCATAGACTTTGAACCAATGCTTGGAGCACCGTTGAGTGCGAGAAAAAGGCTTCAATTTAATATATTCATTATGCCAGTTGACAAGTTTAAACTTATGAAAACATTCCCAAATGCTCTCTTGCCATTGTTTTGGGTTGAGGAAGGTCTTATCTTAGATGACGAGTACCTTAAGCCAATTAAATTGGTATTTACTATGCTAAAGGTCGTCGGAATTATGAAATGGCTAATGATGACAGCAGGAGTAGGATTAGGTGGTGGTGCTGGGTTTCTGTTTTGGAAATCAACACAATCTCCGCAAAAACTTGACATTACTAAGGTTTCGCCAAAAACTATACAAAATGCCGCTGGCGATGAGAAAAAATGGCCGACAAGTGTTAGCACTATTCAAGGAAATAATGCTCCACCCTCTGTAGAAGCCTGA

>*MdemSNMP1*

ATGAAGGAAATGTGGGTAAAAGTACCTTTTCCAGTAGATTTTAGAATTTATTTATTTAATATTACTAATGCAAATGAGATAAAAACTGGTGCTAAGCCAATCGTCCAACAAGTGGGTCCATTTTTTTATGATGAATGGAAAGAAAAAGTTGATTTGGTAGATCGTGAAGAGGACGATACTGTTGAGTACAAAAATAAAGCAACATGGGTATTTAATCAAGCAAAGAGCGCACCTGGTCTCACTGAAGATGTTGTATTAGTTTTTCCTCATGTTATGATTTTATCAATGATCCTCGCGACTGTCAGAGAAAAACCAGCGATGGTTGGTCTTGCGGCCAAAGCTGTCGATAGTATATTCCATAAGCCAGATTCAGTGTTTGTCACAGCGACAGCACGTGAAATACTTTGGACAGGTTTACCAGTTGATTGTTCGGTCAAAGATTTCGCTGGTAGCGCAGTGTGTGGAATACTTCGTGAAGATGATTCAGGATTTTTAAAAGACGGTGAAAATTATAAATTTGCATTATTTGGCGCTAAAAATGGAACTGTTATACCTGACACAATTAGAGTACACCGTGGCAAAAGAAATTATTTAGAGGTAGGGATAGTGACGGAGTTTAAAGGTGAACCGAAATTAAATGTCTGGCCTGAAGAAGGGGACTGTAATACTTTCAATGGCACTGATTCAACTATTTTCCATCCTTTCCTTTATCAAGACGAAGATGTCGTATCTTTTGCGCCTGATCTTTGTAGAAGTTTGGGTGCGAGATATCAAAGACCCTCTAAAGTTAAAGGAATTAAGACAAATCGGTATACCGCAACCTTAGGTGATATGAGTACGGATCCAGCTCTCAAATGTTTCTGTCCAACACCGGATACTTGCTTAGGAAAAGGATTATATGACATATTCCCTTGTGTAAAAGCACCACTTGTCGGTAGTTTACCACATTTTTATGATACTGATCCACAATACTTAACTCAGGTTGATGGTCTTCACCCTAATGAGGAGGACCATCAGATTTTCATAGACTTTGAACCAATGCTAGGAGCACCATTGAGTGCGAGAAAGAGGCTTCAATTTAATATATTCATTATGCCAGTTGAGAAGTTTAAACTTATGAAAACTTTCCCAAACGCTCTCTTGCCATTGTTTTGGGTTGAGGAAGGTCTTTTGTTAGATGACGAGTATCTTAAGCCAATTAAAATGGTATTTACAATGTTAAAAATTGTCGGAATTATGAAATGGCTAATGATGACAGCAGGAGTAGGATTAGGTGGAGGTGCTGGGTTTCTGTTTTGGAAATCAACACAATCTCCACAAAAACTTGACATTACTAAGGTTTCACCAAAAACTATACAAAATGCCGGTGGCGATGAGAAAAAATGGCCGACAACTGTTAGCACTATTCAAGGAAATAATGGGCCACCCTCTGTAGAAGCCTGA

>*NvitSNMP1*

ATGGCTCTCACGAAAATCCAAAAGATCGGCGTCGGCGGCATCTGCATGTTCATATTTAGTTTTCTCTTCAGTGGTGTTATACTGCCGCCTATAGTGAAGCACGAAGTGAAGAAGAAAGTGGCTCTGAAGCAAGGCTGGATGATGAGAGAGGTGTGGGGCAAATTTCCATTTTCATTCGAGTTTCATTTCTACATGTTCAATGTAACGAATCACATGGATATCAAAGGGGGGGCCAAACCCATTGTCGCAGAAGTTGGTCCTTTCGTTTACGAAGAATGGAAAGAAAAGGTAAATCAGGTGGATCACGACGAAGACGATACCATCTCGTATAACGCGAAAAGCACCTTCATTTTTAATGCTGAAAAAAGCAAAGGTCTCACTGGCGAGGAAGAAGTCATAATGCCTCATTTTTTCATACTCGGAACAGTCAACAGTGTGTTGAGAGATAAAGCTAGCGCCATGCCTATCGTTTCTAAGGCCCTCGACAGCATATTTAGAAAGCCAGACAGCATTTTCGTGAAAGCAAAGGTCAGAGAAATACTATTCGATGGCATCGTGATCGACTGTAACGTTAAAGACTTTGCGGGTTCTGCTGTGTGTAATGAAATAGCGCAGAACTATGAAGAGTTTCGTTTGCAATCTATCGGCGACAATAAATACTCCCTGTCTCTGTTCGGTTTGATCAACGGCACGGAAAACAAAGCCCGACATCGCGTGAAGAGAGGCTTGAAGAATATCATGGAGGTGGGGAAAGTCGTCGAGTACGACGGAAAAAACAACGTCTCAGTATGGGACAATGAAATCTGCGACGCGTTTAACGGTAGCGACGGTAGCGTTTTTCATCCCTACTTCGACAAGAAAGGAAAGGACGATCTCGTTGCCTTCAATGCTGACCTCTGTCGGAGTGTCATTTGTCATTACGATTCGGATACTAAATTTGCCGGTCTCAAGTTGCTGCGGTACACGACGGATTTGGGTACCGATGTAGAAAAGTATCCGCACCACAAGTGTTACTGTGTGACACCAGATAGATGTCCGAAAAAAGGCGCCATGGATATCTTCAAATGCGTCAACGCACCGATTATGATCACGAACCCGCACTTTTACCTCGCCGATCCATGGTATGTCAGCGCGATCGAGGGCGTCAAACCGGATAGGGAGAAGCATATGATAATGATCGATATCGATCCGTTCACCGGTTCGCCGATTCACGTCCATACGAGAGCGCAGTTCAACATGTTCCTCCAACCAGTGGAAAAGTTCAAACTGATGAAGACTTTCCCCAATGCTTTGCTGCCACTCATATGGTTCGACGAGATCCTGATTCTGCCAGATTTTCTGCTGAAGGAAATTAAGGGTGGTCATCGTCAAGTTGCAATGGCGAAGGTATTCAAATTCCTCATGATGTTCGGAGGACTGGGAATGGCTGGATATGCTGGATTCATGCACTACAAGGCTACTCAAGGTGAGAACACAACGGAGGTGAAAAAAGTGCCGGTGAAGAGCAGCCCCAACGGTGTGGGAAGTGGCGAAAAGAAAATTAATATCAGTACTATACAACCGGCGCCGCTTCCGCCCAATGTTGACTGA

>*AmelSNMP1*

ATGCGTTTCAAAAAATTGATACATGATATAACGACTCGATTTTTCCATCGAGGAGTCCATGTTATCCTGCATTCGTCGTTTGTGAAACTGCAATCGTTTGTAACAACCGGAAGAAAGCTTCATGAAACTCTTCGTGTACCCGTCGGACGCAAGTGTGTTACTATGAAGCCGAAAAAATTAGGAATCATTGGCGGTTCATTGCTTGCTTTTGGAATTTTGATCTGTGCAATTGCCTTTCCTCCATTTCTGAGATCACAAGTGAAAAAACAAATAGCCTTGAAAGATGGATCCGAGATGCGGGAATTATGGTCCAATTTCCCTGTCCCTTTGGACTTTAAAATTTATCTTTTCAACGTGACGAATCCCATGGAAATTACAGCGGGGGAGAAACCAATTCTCGAGGAAGTAGGACCATTTTTTTACGACGAGTACAAACAAAAGGTGGATCTGGTCGATCGAGAAGAAGACGACAGTTTGGAGTACAACTTGAAAGCGACGTGGTTCTTCAATCCATCTCGAAGCGAAGGATTGACCGGTGAGGAAGAGCTGATCGTTCCTCACGTTCTCATTCTGAGCATGATCAAACTCACCCTCGAGCAGCAACCGGCCGCCATGGGGATTCTCAATAAGGCTGTGGATAATATTTTCAAGAAACCAGAATCCGTGTTCGTAAGAGCGAAGGCGAGGGAAATACTTTTCGACGGTCTACCAGTTGATTGCACGGGAAAAGATTTCGCCTCGAGCGCAATTTGCAGCGTTTTGAAAGAAAAGGACGACGCTTTGATAGCCGATGGACCAGGACGTTACTTGTTCTCACTCTTCGGCCCTAAAAATGGCACCGTTCTTCCCGAACGTATACGAGTGTTGAGGGGTATAAAGAATTACAAGGACGTGGGGAAGGTAACCGAAGTCAATGGAAAAACGAAGTTGGACATTTGGGGAGAGGGCGATTGCAACGAATTCAATGGCACAGACTCGACGATCTTCGCTCCATTGCTCACGGAACAAGACGATATTGTCTCATTCGCTCCTGACATTTGCAGAAGCATGGGGGCCCGTTTCGATTCATACACAAAAGTCAAAGGCATCAACACGTACCATTACAAGGCTGATTTGGGCGACATGAGCTCACACCCGGAGGAGAAATGTTTCTGTCCCTCTCCAGATTCTTGTTTAACGAAAAATCTCATGGATTTGACCAAGTGCGTAGGTGCTCCTCTCATAGCTTCCCTCCCACATTTACTTGGTGCTGAAGAAAAGTATCTAAAAATGGTTGATGGACTTCATCCGAACGAGGAAGAACACGGAATCGCCATGGACTTCGAGCCAATGACAGCTACTCCCTTAAGCGCGCACAAGAGGTTACAATTTAATTTATATCTTCACAAAGTTGCAAAGTTCAAATTAATGAAGAACTTCCCGGAATGTTTGTTCCCAATATTCTGGGTCGAGGAAGGAATACTGTTGGGTGACGAATTCGTGAAGAAATTGAAGACCGTGTTCAAGACAATAAGCATAGTCGGATTTATGAAATGGTTCACAATAGTAAGTGGTACTTGCGTGAGTGGAGCAGCTGCCGCGTTATTCTTCAAGAACAAAGATAAAAATAAACTGGACATCACAAAGGTAACGCCGCAAAAAGGTGAGGAGAAGAAATGGCCTAACCAGATGACTATAAGTACGATACAAAGCGCAGCAGTACCCCCTAATCTCGACGCGGATTAA

>*AgamSNMP1*

ATGGAGCTCAAGGAAAGAAATTTTAAAAAGATTGGACTTATCTGCGTCGCGGTGCTTCTGTGTGGTATGGTGTTTAGCTATGGGATATTTCCGTCGATTTTGCGTTTCATGATAAAGCAGAACGTGCTGCTGAAACCGGGCACACAAATACGGGACATGTTCGAGAAGATACCATTTCCGCTCGACTTCAAGCTGCACATCTTCAACGTGACGAACCCGGATGAAATTATGCGCGGTGGCAAACCGCGCGTCAACGATATTGGACCACTTTATTTCGAAGAATGGAAGGAAAAGTATGACACGGTGGACAACGTGGAGGAAGACACGCTCACGTTCACACTGCGCAACACATGGATATTCCGGCCCGATCTGTCCGCCCTAACTGGCGAGGAAATTGTCACCATACCGCATCCACTCATTATGGGCGTTCTGCTGATGGTGCAGCGGGACCGCGAAGCAATGATGCCGCTGGTCAAGAAGGGCGTCAACATCCTGTTCGATCCGCTCGAGTCCGCCTTCCTGAAGGTGCGCATCATGGACCTGCTGTTCGATGGCATTTACGTCGACTGCAGCAGCCAGGACTTTGCTGCGAAGGCGCTCTGCTCCGGCATGGATTCAGAAGGTGCAGTTATGCCGCACAACGAAACCCACTACAAGTTTTCCTTCTTTGGCATGCGCAATCACACCGAGGCAGGCCGTTGGGTCGTGTACCGGGGCGTGAAGAACATTCGCGACCTCGGCCGGGTGGTATCGTACAACGAGGAAACGGAGATGGACATCTGGGACGGGGACGAGTGCAACCAGTACATCGGTACCGATTCGACCATCTTCCCCCCGTTTCTCACCGCTCAGGATCGGCTATGGGCATGGTCGCCCGAAATCTGTCGTTCGCTCGGTGCGCACTACGTGCACAAGTCAAAGTATGCCGGGCTGCCGATGAGCTATTTCGAGCTGGACTTTGGCGATCTTAAGAATGAGCCGCACAATCACTGCTTTTGTCGCGATGCGCCCGACGATTGCCCACCGAAGGGCACGATGGATCTGTCGCCCTGTCTTGGGGGACCCATAATCGGCTCAAAGCCCCACTTCTACGGCGCCGATCCGAAGCTGGTCGAAGCGGTGGACGGGCTGGCACCGAACAAGGCAGCACACGATGTCTACATTCATTTTGAGCTGGCAAGTATTTGCTGGGTTTCTCCGGTTTCGGCCGCAAAACGGCTACAGTTCAGCATGGAGCTCGGCCCGATACGAGACCACGAGCTGTTTGGTCAGCTGCCGGACGTAATTTTGCCCATGTTTTGGGCCGAAGAGGGTGCCTCGCTCAACAAAACCTGGACCAATCAGCTGAAATATCAACTGTTTCTGGGGCTCAAGTTTAATGCCACTGTCAAATGGTTAACCATCATCATCGGTACGGTCGGGGCCGTCGGGTCGGCGTACATGTACTTTCGCAAGGAAACCAAAACAACCGACGTGGCCCCGGTGGATGTATCGACCCCAGACACCAATCCGTCGTCTGCCAAGGATGGTGTTGTGAACGTTTCGCTCGGCAGAAACCTGCCGCCCGTTATCGACGGGTTGGACAAACCGCCGAAGCTCAGGGCGACCGAGCTTCAGCAGGAACGGTACTAG

>*AaegSNMP1*

ATGCTTATTAAGAACCGAAAGAACTTGATGCTGAAGCCGGGCACTCAGATGCGAGGAATGTTCGAAAAAATCCCGTTCCCTCTAGACTTCAAGCTGTACCTGTTTCACGTTACCAACCCGGACGTAGTGATGAAGGGTGGCAAACCCCGTGTTCGAGAGATTGGACCGTACTTTTTCGAGGAGTGGAAAGAAAAGTACGACACCGTGGACAATGAGGAGGACGACACGCTCACATTCACGCTGAAGAACACCTGGATTTTCCGCCCGGATTTGTCCAAGCCGCTGACGGGAGACGAAATGATCACCATTCCGCATCCGCTCATCTTGGGAGCTTTGCTGATGGTCCAGCGGGATCGGGAAGCCATGATGCCGTTGGTTTCCAAGGGCATGGACATCATCATGAACCCGTTGACGACGGGTTTCTTGACAACTCGAGTTATGGATCTGCTGTTTGATGGGATTCTGATCGATTGTAGCAGTCATGAGTTTTCGGCGAAGGCCTTATGTTCGGGGCTGGAATCGGAAGGCGCTGTCATGCCCTTCAATGAGACTCATTTCAAGTTTTCGATGTTTGGATTGAAAAACGGAACCGATGCCGGTCGATGGGTGGTCTACAGAGGGGTCAAGAACATCATGGATTTGGGTCGCGTCGTGAGCTTCAACGATGAAACGGAAATGGATATCTACGACGGGGATGAGTGCAACCGGTACATCGGAACGGACTCGACCATCTTTCCGCCGTTTTTGACAACGAAGGATAAGCTTTGGGCTTGGTCACCGGAAATCTGTCGCTCGATCGGAGCGGAGTATGGGGGAAAGTCCAAATATGCAGGGCTGCCGATGAGCTTCTTCAAGCTGGACTTTGGCGATGCAAGGAACGAACCGGAACATCATTGTTTCTGTCGTGACCCACCGGACATCTGTCCACCGAAGGGAACAATCGACCTGGCTCCTTGTCTGGGAGCACCGATCATTGGTTCCAAGCCGCACTTCTATGACTCTGACCCGAAATTGTTGGCTGCCGTAGATGGACTGACGCCTAACGAGAAGGACCACGATGTGTACATTCATTTCCAGCTGCTTTCGGGTACTCCGGTATCGGCTGCCAAGCGGCTGATGTTCAGCATGGAAATTGAACCCATTAGGGACCATGCGGTATTGGGAAATCTGCCGACCGTTATCTTGCCGCTGTTCTGGGCAGAGGAAGGTGCTTCGCTCAACAAAACCTGGACTAATCAGCTCAAGTACACCTTGTTCCTTGGCCTCCGGTTCAACACTGCCGTCAAGTGGTTGACCATCATCATCGGAACTATCGGAACCATCGTGGGTGGGTTCATGCACTACAAACGGACGACCAAGATGGTTAACGTCACTCCGGTACAATCCGTCAACGGAAGCAGCGCCAAAGGCAAGGGAGCTGGCATGACAGTGGTTGGCCATCAACCGGATAGCAAAGGTGGGTCCGTGACGGCACCGGTCATACCGAGTGCTAAGGATCTGCTGCAGAACAGTCGAAACTTGCCGACAGTTATCGAGGGACTGGACAAACCGCAGAAAGTTACGGTCACTGAAATGCAGGAGCGGTATTGA

>*DmelSNMP1*

ATGCAAGTACCTCGGGTTAAGCTGCTCATGGGGTCGGGAGCCATGTTCGTGTTTGCCATCATCTATGGCTGGGTTATCTTTCCCAAAATTCTCAAGTTTATGATATCGAAGCAAGTAACTTTAAAGCCCGGATCGGATGTTCGGGAACTCTGGTCCAATACTCCATTCCCACTGCATTTCTATATCTACGTTTTCAACGTAACCAATCCGGATGAGGTATCCGAAGGAGCTAAGCCACGGCTACAGGAAGTGGGTCCCTTCGTTTTCGATGAGTGGAAGGACAAGTATGATTTGGAGGATGATGTCGTAGAGGACACGGTCAGCTTCACCATGAGGAACACGTTCATTTTCAACCCGAAGGAGTCGCTGCCACTTACGGGCGAGGAGGAGATTATTTTGCCGCATCCCATAATGCTGCCTGGCGGCATTTCGGTTCAAAGGGAAAAGGCTGCCATGATGGAGCTGGTTTCCAAAGGATTAAGCATTGTCTTTCCGGATGCCAAAGCCTTTTTGAAAGCTAAATTTATGGATTTATTTTTCCGCGGCATCAACGTAGATTGCTCCTCGGAAGAATTCTCCGCAAAGGCGCTCTGCACCGTGTTTTACACGGGAGAAATTAAGCAGGCCAAACAAGTCAATCAAACTCACTTTTTGTTCTCGTTTATGGGTCAGGCCAATCACTCGGATTCTGGCAGATTCACTGTCTGTCGCGGAGTGAAAAATAACAAAAAGCTTGGAAAGGTAGTGAAATTCGCCGACGAACCGGAGCAAGACATTTGGCCAGATGGCGAGTGCAACACCTTTGTGGGCACTGATTCTACGGTCTTTGCACCGGGCTTGAAAAAGGAGGATGGCCTGTGGGCATTCACGCCGGATTTGTGTAGATCCCTGGGCGCTTATTATCAGCACAAGTCATCCTACCATGGCATGCCATCGATGAGATACACCCTGGATTTGGGGGATATACGTGCCGATGAGAAGCTTCATTGTTTCTGCGAAGATCCCGAAGATCTGGACACGTGTCCCCCGCCCAAGGGCACTATGAATCTGGCGGCCTGTGTTGGTGGTCCATTGATGGCATCAATGCCTCATTTCTATCTGGGCGATCCAAAGCTGGTTGCCGACGTTGATGGTCTCAATCCGAATGAAAAGGATCATGCGGTCTACATAGACTTTGAACTTATGTCTGGAACACCTTTTCAGGCTGCCAAGCGTTTGCAGTTTAATCTGGACATGGAGCCAGTGGAGGGCATTGAACCCATGAAGAATCTACCCAAGCTGATATTGCCCATGTTTTGGGTGGAGGAGGGCGTGCAGTTGAACAAGACCTACACGAACTTAGTGAAATACACACTATTTCTGGGCCTAAAAATCAACTCAGTCCTGCGCTGGTCCCTCATTACTTTCTCCCTGGTGGGCCTGATGTTTTCCGCCTATCTTTTCTACCACAAATCCGACAGTTTGGACATAAACAGCATCCTCAAGGATAACAACAAGGTAGACGACGTGGCCAGCACAAAGGAGCCCTTGCCTTCAGCAAATCCAAAGCAATCTTCTACTGTGCACCCTGTTCAGTTGCCCAATACTCTGATTCCCGGCACGAATCCCGCTACCAATCCAGCTACCCATCACAAAATGGAGCATCGGGAGCGCTACTAA

>*DpseSNMP1* ATGAAACTCGATCGCATGAAGCTGCTCTTCGTCTCCGCGGGCACCCTCGTCTTTGCCATCCTCTTCGGCTGGGTTATGTTCCCAAAGATACTTAAATTTATGATCTCCAAGCAAGTAACGCTAAAGCCCGGCACGGATGTCAGGGAACTCTGGTCGAATACCCCCTTCCCGCTGCACTTCTATTTCTACGTTTTCAATGTCACCAATCCGGAGGATGTATCCCAGGGTGGACGGCCACGGCTCCAGGAAGTGGGTCCCTTTGTGTTCGATGAATGGAAGGACAAGATTGATTTGGTGGATGACGTTGTGGAGGATTCGGTCACCTTTACCATGAGGAATACTTTCATCTTCAATGCGGAGGCATCGTATCCCCTGACGGGAGAGGAAACTATTACCCTGCCACACCCCATCATGCAGCCTGGCGGCATTACAGTCCAGCGAGAACGTGCGGCCATGATGGAGCTGATAGCCAAGGCCATGTCTCTGGTGTTTCCCGGGGCCAAGGCCTTCCTCTCCGCGCCGTTTATGGATCTGTTCTTCCGTGGCATCGATGTGGACTGCTCCCCGGATGACTTTGCTGCCAAGGCCCTCTGCACGGTGTTCTACACGGGCGAAGTGAAGCAAGCGAAGCAGGTCAACCAGACGCACTTTTTGTTCTCATTCATGGGCCAGGCCAACCACTCGGATGCTGGACGCTTTACTGTCTGCCGGGGCGTGAAAAATAACAAGAAACTGGGCAAAGTCATACGATTCGCCGAGGAGACCGAAATGGATGTCTGGCCGGGCGATGAGTGCAACCAGTTCGAGGGCACCGACTCCACCGTGTTCCCGCCTGGCCTCAAAAAGGAGGAGGGACTGTGGGCCTTCACCCCCGATCTGTGCCGCTCTTTGGGGGCCACCTATGTGAGGAAATCCTCGTACCATGGCATGCCCTCCACTAGATACACTCTGGACCTGGGGGACATGCGTTCGGAGGAGAAGCTGCATTGCTTCTGCGATGATCCCGAGGATCTGGACACGTGTCCCCCGAGGGGCACCATGAATCTGGCCCCCTGTGTGGGGGGCCCACTGTTGGCCTCCATGCCGCACTTTTACAATGGCGATCCAAAGCTCGTGGCGGCCGTTGATGGCCTCCATCCGAATGAGAAGGATCATGCGGTTTATATCGACTTTGAGCTGATGTCTGGCACTCCCTTTCAGGCGGCCAAGCGTCTCCAATTCAATCTCGATATGGAGCCCGTGGAGGGCATTGAGGCGCTCAAGAATCTCCCAAAGCTCATCCTTCCACTCTTTTGGATCGAAGAGGGCGTCCACCTGAACAAGACCTACACGAATATGGTCAAATACACGCTATTCCTTGGCTTAAAATTCAACTCTGGTCTGCGTTGGACTCTCATTACTCTCTCCCTGGTGGGTCTCATGTCCGCTGCTTATCTTTTCTATCAGAATTCCGATAGCCTGGACATCACTCTGCCTCCGAAGATCCTCAAGGAAGTCAACAAAGTGGCCGATGCGGCCATGAATTCGAAAATGTTCCCTGAGAAAGCTCCCACCACTCCACAGACGACTATTCCGGGCACCAATCCACCCACCAATCATGGCGCCCAGCCACCTCCTGCTGTGGCTTCGGTTCCAGGCATAATTCCGCCATTAAGTCTCAAAATGGAGCAAGCACAGCGCTATTAG

>*BmorSNMP1* ATGCAGTTGGCTAAGCCGCTTAAATATGCCGCAATCAGCGGTATTGTCGCGTTCGTGGGTTTGATGTTCGGATGGGTGATATTTCCGGCCATTTTGAAGAGTCAGCTTAAGAAGGAAATGGCGCTATCCAAGAAGACGGACGTTCGGAAAATGTGGGAAAAAATACCTTTCGCACTCGATTTCAAGATATATCTATTTAATTACACTAACGCTGAAGATATTCAGAAAGGAGCTGTACCGATCGTGAAGGAAGTTGGGCCTTTTTATTTTGAAGAATGGAAAGAGAAGGTAGAGGTTGAAGAAAACGAAGGAAACGATACGATAAATTATAAGAAAATCGACGTATTCCTCTTCAAACCCGAGCTGTCTGGTCCAGGACTGACGGGAGAAGAAGTCATCGTGATGCCTAATATCTTTATGATGGCTATGGCGCTTACCGTTTATCGAGAAAAGCCTGCAATGCTGAACGTCGCTGCTAAAGCTATCAATGGAATCTTCGACAGCCCAAGCGACGTCTTCATGAGGGTCAAAGCCTTGGACATCCTCTTCCGCGGCATAATCATCAACTGTGATAGAACAGAATTTGCTCCGAAAGCCGCCTGCACCACGATAAAGAAGGAAGCGCCTAATGGAATTGTTTTCGAACCAAATAATCAGCTTAGATTCTCTCTATTTGGTGTGCGTAACAATTCGGTGGACCCTCACGTTGTGACAGTCAAACGTGGAGTCCAAAACGTTATGGACGTCGGTCGAGTCGTAGCGATAGACGGGAAAACCAAAATGAACGTTTGGAGGGACTCTTGTAACGAGTACCAAGGAACGGATGGCACCGTTTTCCCGCCATTCCTGACGCACAAAGATCGCCTGCAGTCATTTTCCGGGGATTTGTGCAGGTCATTCAAACCGTGGTTTCAAAAAAAGACATCGTACAATGGGATTAAGACGAATCGCTATGTCGCCAACATTGGTGATTTTGCCAACGATCCAGAATTACAGTGCTACTGTGACAGCCCTGACAAATGCCCCCCAAAAGGCCTGATGGACTTGTACAAGTGCATCAAAGCTCCAATGTTTGTCTCCATGCCTCATTACTTGGAGGGAGACCCGGAACTGTTGAAGAACGTGAAAGGATTGAATCCTAATGCTAAGGACCATGGAATCGAAATCGATTTTGAACCGATCAGTGGTACGCCGATGGTTGCTAAGCAACGCATTCAGTTCAACATACAGCTGCTGAAATCAGAAAAAATGGAATTACTCAAAGATCTTCCTGGAACTATTGTTCCTTTATTATGGATTGAAGAAGGTTTGTCATTGAACAAGACATTCGTGAAAATGCTTAAAAGTCAGCTGTTTATACCGAAGCGTGTTGTCTCCGTGGTTTGCTGGTGTATGATATCTTTTGGCTCTGTTGGGGTCATAGCCGCAATGATATTCCATTTCAAAGGTGATATAATGCACTTGGCTGTGGCAGGAGACAACTCAGTTTCCAAAATAAAACCTGAAAACGAAGAAAATAAAGAAGTGGGCGTTATGGGCCAGAATCAAGAACCAGCCAAAGTGATGTAA

>*TcasSNMP1*

ATGAAACCGATTCGTCGGAAGGACTTCTTATTTTTACTTTTTCCTAAGTTGCGTTTACTTAAGTTCTCTTTTGATTCGTTAAAGTGCATTTATCATTGTTATACGAAGAAGAAAACTAAAAAGGAAATGAGGTTGCCGGTCAAAATCGCGATAGGATGCGCGATTGGGCTAGTTGTTATCATTGTTTTCGGATTTATTGCTTTTCCTAAAATGATCAAAGGCAAAGTGAAGAGTATGATAAATTTGAACAAGGGAAGCGAAATCCGCCAAATGTTCGTCAAAGTCCCGTTCGCTCTAGACTTTAAAATTTATATGTTTAACGTAACAAATCCGATGGATGTCCAGAAGGGTGCTTTGCCTGTCCTAAAGGAAGTGGGACCGTTCTGCTTCGAGGAATGGAAGGAGAAGGTTGACCTTGACGATAACGACGACGAGGATGTCATGTTTTACAATCCCAAGGACACGTTTTACAAGGCCAACGGGCCTGGATGCCTCGACGGAAGCCAGATGATTACAATGGCACATCCTTTAATACTCGGGATGGTGAACACAGTGGTGCGGACTAAACCAGGCGCCATTTCCTTGATTAGCAAAGCCATTAATTCCATTTACGGCAATCCTGATTCTATTTTTATGACGGCTTCGGCAATGGACATCCTGTTTGATGGTGTTGTAATTAAATGCGGGGTCAAAGATTTCGCCGGAAAGGCCGTTTGTTCGCAGCTGAAAGAAGCACCAGACTTGAGGCATGTCGACGAAAATGATTTGGCGTTTTCATTTATAGGGCCTAAAAATGCAACTCCCGGGAAGAGATTTAAAGTTCTGAGGGGCGTCAAGGAATCGCACGATGTGGGACGGATTCTCGAGTATGACAATAAGAAAGAAATGGAAGTGTGGCCTACGAAAGAATGTAACCAATATAAAGGAACAGACGGGACGGTATTTCCCCCTTATCTCACAAAGGAGGAAGGTCTTGCCTCCTATGCGCCCGACTTGTGCCGTTCGTTAGTTGCCGTCTACAGTGGGGACACAAAATACGACGGTATTCCTGTACGAATATACACGGCGACTTTAGGCGACATGTCCAAAAATGCAGACGAGAAGTGTTACTGCCCCACACCTGACACTTGTCTGAAGAAGGGCATGATGGACCTGTTCAAGTGTGCAGGAGTTCCTGTCTATGTCTCACTACCACATTTCTACGAATCGGACGAAAGCTACGTCAAAGGAGTCGTCGGCCTTAACCCTAACAAGAAAGACCACGGAATTCAGATTTTGTTCGAATCGACAACTGGAGGTCCTGTCAAGGCCGCGAAACGCCTCCAGTTCAACATGCCCCTGGAGCCGAATCCAAAATTGCCAATTTTTGCAAACTTGCCCAACACAGTTTTGCCACTGTTCTGGGTGGAGGAAGGCGTGGCGCTGAATAATACGTTTACGAAACCGTTGAAAGATCTTTTCAAAATAATGAAGATTGTAAAGATCGCGAAATGGTTGATTATGCTGGGATGTTTGGGGGGTTTGGGAGCCGCCGGTTATCTCTACTTTTCCAAAAAAGGGGAAGCGAATATTACGCCGGTACATAAGGTTAAACCGGCCGAAAATGGGGTTAGCACTCTCGGGGGTGAAGTCAACCATGCCATGTCTGATAACGAGATAGAGAAATACTGA

**Supplemental material 3**

The genomic sequence of MmedSNMP1

>MmedSNMP1 genomic sequence

ATGCTTTTATTCAAAAAACTTGGTATTGCCGGTGGTTCGGTATTTACTTTAGGTATAATAATTGGTTACGCGTTTTTCCCGCCATTTTTAAAGAGTCAAATTAAAAAGGTATATTCTTACTATTCCGATAATTAATTATATTAATTATCGACTAATATTTCATTTAAATTATTTTATAAAAATAGTGATGTTGATTTTTAAAAAAAATATGAGATTGTCAATTCATCAGACTTAACTCTTGTTCATGTGTCATGTCACACGTTATATAGTAATTTATGTATCAGTGTCATTACTGCAATTATTTTCGAATACTTCCTATCAAAATTACATAACACAGTGCAAAAAAAAATTAAATTACAGCTAATAGTTGTAATAACAAATTATTATATTTTATTACCACAATATGTTTTTATAAACAAATTGTATACATAAGCAGTATATATGAACAAATAAATATTTTATTTTATGTGATTTAAAGAAGCAAATAGTTAGATCATCATGTTATTAATTAATAAGTATAAACTTATTTAATAATCAGTCTGACGATGACAGTTTCACGGTATCGTGATTAGTTTTTTTTTTTTTTTTTTTTTTGTAATTAAACGTGTATACAATAATCGGTATCTTCATTATTTACTTGCTATTGAATTAAACATTTTTTTTACTCCGGTTTTTATTGAATATTCTTAGCAGCCATTAAGATAAAATCACGTGGATTATAAATAAAAAAATATCATTTGCTTCTTTTAGTATACATTGTAGTTTTAATGAGCGTTAATAAATAAATATGCAAGTCATAAATGAGGAAAATTTTATATAATTAATTTTATGAAAATATTTACAATCGTAAAATGTTTATTTATTGGTAGTATGGATTATTAATTATTTAATTATTCATATTTTTTCTTTAATATCGTAATGTATTCGATAGGGAATACAATTGGTCGATGGCTCAGACATGAAGGAAATGTGGGTTAAAGTACCTTTTCCACTAGACTTTAGAATTTATTTATTTAATATTACTAATGCAAATGAGATAAAAGCTGGCGCTAAACCAATCGTCCAACAAGTGGGCCCATTTTTTTATGAGTAAGTGTAATTTAATTTCATAAATTTTTTTTTTAAATATGATAGAAGAATCCGGGGCAAAACTAGACACTATTCGAAAGTCGAAAAAAATCTTTTTTTGTATAAAAATGTTTTTTTGTCCGGTTAAGTGGAAATTGAGGTAAGTTGAGCATCCTTAAGGCTGCTATTTCAAAATTTTTACCATCAATAATTTTGTCACTGCCTGGGGTGGGCAACTTGCCTCAATGTTTCCGAACCACTGTAAATTACACTGCACATGGAGAAAATTTAATTATACTCTCTACGATGTTAAATTTTTAATTTAAACTATTTGAAATGGGAGCAAAATTTTACGTAGAATAATAATTAGTTTTACCATTGAGAATGATAACAGTAACAATCGATGGTCGTGACGATTACTATCGAAGATGCTAATTGTAACTAATAAAGATTACAATTGTTACAATCGAAGATGGCAACTGTTACTATTCAAGGTTGCAAAAATTCTGAAACAAAAAATACGATAAATTTATATACATTTTGTCTTTTAAACAATTAAGAGGATTTGCCATCTATTCTCTTTTTCACGCGCATGTGAACAAACTAAACGGTCCTTGTCACACTTACATTAGGAAATGGAAATTTAAAATGAGCCGTTCCCAGACATAAACTGAAATTTCCGAACCAGATGCATTTCATTCGATAAATAATATCTTAACGCATCGAATGAAATATTTTATTTAATGATTTGAGTTTTTATCTATGAAAATGGTTCATTTGAAAATCACCTATTCCACTTTTTTAAAATGAGTATCAAAACAATCGTTGACCGTATAGGGTAAGAGCACCAGTCCCCAGCCCTTAACCAGTAGCCAGCCGATCATATACTTTAACATTGGCTTAAAATATATATAGATATAATTTAACATGAGGTGGTTGGCTACTGGTGCTCTTACCCTACAATAAAAATTTCCAAATATCGGAGCTGTTATTAATAACTTTAAATAATTGGATATGATCATGAAAATAGCAATCATCCAAGTATTGAATGATGAAGAAAACTTTATGATGATAATCATCAATAATCAGATTTCTTTGCAACATAAAACAATAGGCCATCATTTGATCTGTAAAGCCGGTATTTAAATTCTTTATTAGTTTAGATAGTAATTATTATTTGAGCTGATGGTAGCTGTTACTATCAGGTTATTAAGAATTTCAATGGCGATAATGATAATAGTTACAATTTCTGATTATCTAGACTAAACGATAACTTTTACTGTAAAATTCTCTCCGTGTACTAATAATTTTTTTTAAAAATGATGTGTCAGTTTACCTCGAGTTCCCTAAATATATGTAATGTCACTAATGATCCTGAAGTTAGCAGACAATCAGCAATTTTTGGATTTTTATTTTAAATAGGTCGATTACAAAAAAAAAAAAAAAAAAAAATGCACATGTAGAGAATTTAAAAAACTATAAGTGCAATTTTTTCAAATATTTTTTTTTACGATTTGTCGAGTTTAAAAAAATCTAAAAAATATCAGACGTCGGCTAACTTCAGTATCATATGTCACTATACTATAATAACTTTTGTTTATAATTATAAAGGGAATGGAAAGAAAAAGTTGATTTGGTAGATCGTGAAGAGGACGATACTGTTGAATACAAAAATAAAGCAACATGGGTATTTAATCAAGCAAAGAGCGCTCCTGGTCTTACTGAAGATGTTGTATTGGTTTTCCCTCATGTTATGATTTTGTCAATGATACTCGCGACTGTCAGAGAAAAACCAGCGATGGTTGGTCTTGCGGGTAAACTACTAATTGTGAAAATACAAAAATTATTTTCTGACGATCTAGAAGTGCTTTCTTATGACGAATCAATGAAAAGTATTCCAAACTATGAATATTTATTGGTTGTCAGTACTTTTGACGGTTTCATTTTTAGAGAAACTTCATCTAATTTTTTTTTGTAAAACCACTTCTAGAAGTCGCATCTTTGAAACTCGTGGATTTATGATATTTTTATTACAGCCAAAGCTGTCGATAGTATATTCCACAAGCCAGATTCGGTGCTTGTCACAGCAACAGCACGTGAAATACTTTGGACAGGTTTACCAGTCGATTGTTCGGTTAAAGATTTCGCTGGTAGCGCAGTGTGTGGAATACTTCGTGAAGACGATTCAGGTTTTTTGAAAGACGGCGAAAATTATAAATTTGCATTATTTGGCGCTGTAAGTAATAATATTTTTATTTATATAAACTATACATATATTAAATATCACCAGCTGTATGCGATGCACTAAAAAATTAGGAATTAATGTAATTTCACATTCACTCCAATTCGAAATTTTAATACTAAAATAAACTCCCATTTGGAGCGAATTTTCTTCTAAGAGAATTAAATAAGTCGCGACACTAATTCCAAAAAAACTTATTATTTTTATAAGGAAATACTTTAAAGCCAAAAGAGCGAATCATTTTTTTTTTCATTGTCAAACTTATTGTAGACTTATTTTTCAGCTAAAAATTTTATTTATAGATTTTCAGCTTTAGAAGAAGTTTACAATATAATTAGAAATTAAATTTTTTAAAAATTATACGCCCTTTTGGTTTAAAACTTAGCGACTAAAGTCAAAAGGACGTATAAAAATATAAATCCATTTGAAATTGATGACGCGAAGTACAGTCATCCGTTCCGCATTCACTCCGGATTTAATCCACGTTCATTCCACAAATTTTTCACAGTGTATATAGCCACTAATATCAAATCAACATATATATATATATATGTATTTATTTGAGCTCTATATCCTTGTACTATTGATTTTCACAATCAAGTATTGCTTAATAAATAACACAATAAAAGTTTTGTAATTGCGTCATTAAATGTAATTTAACATTTTTTTTATTGGATAGAAAAACGGAACTGTTATACCTGATACAATTAGAGTACACCGCGGTAAAAGAAATTATTTAGAGGTAGGAATAGTTACGGAGTTTAAAGGTGAACCGAAATTAAATGTCTGGCCTGAAGAAGGAGACTGCAATACTTTCAATGGCACTGATTCAACTATTTTCCATCCATTCCTTTATGAAGACGAAGATGTCGTATCTTTTGCGCCCGATCTCTGTAGAAGTTTGTCTGCGATATATCAAAAGCCCACTAAAGTTAAAGGTTTCTATGAATTTAATATGTTGATATTCACTTACGCAAAAAATTAAAGGAACAGAAAAATTTCATAAATTTTTTAGTGATTTTTGGAAGGCTGTCGTGAAAAATAATCAAATCGAGATTAAAAAAAGCATTTTATAGCTTGAAATCTCTAATTTCAGTGCATTATTACCATTACAGCTTTCCAAAAATTACTAAAAAATTTATGAAGTTTTTCTGTTCCTCTAATTTTTTGCGTAAGTGTATATTTTTCATAACTATACTTAAATTCTTTATTTGCACATGTGAATATTTATTCTGCTTACTTATTCAGGAATTAAGACAAATCGGTATATCGCAAGTTTGGGTGACATGAGTACGGATCCAACTCTCAAATGTTTGTGTCCAACACCGGACACTTGATTAGGAAAAGGATTGTATGACATATTTCCTTGTGTAAAAGCACCACTTGTCTGTAGTTTACCACATTTTTATGATACTGATCCACAATACTTAACTCAGGTTGATGGTCTTCACCCTAATGAGGTAAAAATATAATGGATTATTTATTTTAAAACAAGTGATTAAATACATACGTTAATATTATGCATGTAATGGTGGTACTTTTCAGGAGGATCATCAGATTTTCATAGACTTTGAACCAATGCTTGGAGCACCGTTGAGTGCGAGAAAAAGGCTTCAATTTAATATATTCATTATGCCAGTTGACAAGTTTAAACTTATGAAAACATTCCCAAATGCTCTCTTGCCATTGTTTTGGGTTGAGGAAGGTCTTATCTTAGATGACGAGTATCTTAAGCCAATTAAATTGGTATTTACTATGCTAAAGGTCGTCGGGTAAGTAAACAGTTCTTAACCATAACTACACCGAAAGTTTAAATTCCTGCCTTGTTTAGAGAGAACAGAAGAATTAGTATACAGACCAGTGGCGTTATGAGTCCGCTGATTTATATTTCTTGGAAAAAATTCCCCCTCTATGACTAGGAAAATAATCATGTATACACTCACGCGCTTGCAAAGTAGGGGAAGTGTGATTTCGGCGTTCAACTGCTGAGCAGAAGTGGGGGTACAGGAATTCTTAGAATTATATTCCCCTACACCCCTGCAGGCGGACTTATAACGCGATTCGACTGTATTACATACCTAGGGAGGAAATTAGGTCATTTTTAAGTCACGTGTGTAATTGGCAAACACGCGGATTAGGACGTCCTGCTTTCTTCCGGGGTGTGTAAATTATTTTTCACTGTATCTGCACCTGAACGTTTAAATTTTGGTCTCTATCTATGGGAAGAATATCGCAGCGCTGATTTCTATCATTCGTTTTTTCATAAAAGAAAAGAATTTCTTCTCTCTGTACATATTCAGAGGCGGGAATTTAAATTTCGACGTAGGTATGGTGAAAAATTGTTCTTTTCGTATGAGAAAACAAATGATAGAAATAAGTACATGCGTTATTCTTTCCATGAATAGAAGCAAAAATTTAAACTTTCAAGTGCGAATTTGATGAAAAATAGTTTATACACCAAGGGGGGAAAGTAGGACATCACAATCCGAATTTTTGCCACCCTTGGGATCAGTTCGAGTAGGCAATTACACACGTGGCTGTGAATGTCCAACTTTCCTCGCTTCGTTATAATTTCAGTGCATCATAATACTCAAAAATTTACAATAATGACAAAATAAATAAAAAATTATTGATAATAATTAATTTATTGCTCCTTTTTTGCCAAATTTTAAAAGTGCTCTTAAACTTGTGCTCCAAATTTTATTTATCCTTTATTTTTGTTTTAATGTAACTAAGTAATGCTTATTTATGCCAACAGAATTATGAAATGGCTAATGATGACAGCAGGAGTAGGATTAGGTGGTGGTGCTGGGTTTCTGTTTTGGAAATCAACACAATCTCCGCAAAAACCTGACATTACTAAGGTTTCGCCAAAAACTATACAAAATGCCGCTGGCGATGAGAAAAAATGGCCGACAAGTGTTAGCACTATTCAAGGAAATAATGCTCCACCCTCTGTAGAAGCCTGA
